# Circulating Vitamin D Levels and the Risk of Atrial Fibrillation: A Two-Sample Mendelian Randomization Study

**DOI:** 10.3389/fnut.2022.837207

**Published:** 2022-03-28

**Authors:** Shengyi Yang, Hong Zhi, Ying Sun, Lina Wang

**Affiliations:** ^1^Key Laboratory of Environmental Medicine Engineering, Ministry of Education, Department of Epidemiology and Biostatistics, School of Public Health, Southeast University, Nanjing, China; ^2^Department of Cardiology, Zhongda Hospital, Southeast University, Nanjing, China; ^3^Zhalongkou Community Healthcare Center, Hangzhou, China

**Keywords:** 25-hydroxyvitamin D, vitamin D, atrial fibrillation, Mendelian randomization, cause-effect

## Abstract

**Aim:**

We performed a two-sample Mendelian randomization (MR) analysis to evaluate the association between serum vitamin D levels and atrial fibrillation (AF) risks.

**Methods:**

Data on the single-nucleotide polymorphisms (SNPs) related to vitamin D, 25-hydroxyvitamin D, and AF outcome were obtained from a UK Biobank study, SUNLIGHT consortium, and the latest meta-analysis of genome-wide association studies GWASs with six independent cohorts, respectively. MR analysis was performed to obtain the estimates, followed by the use of inverse variance weighted (IVW) method, weighted median method, maximum likelihood, MR-egger method, and MR-PRESSO methods.

**Results:**

The IVW estimate showed that genetically predicted vitamin D and 25-hydroxyvitamin D levels were not causally associated with the risk of AF with two models. The association was consistent in complementary analyses.

**Conclusions:**

Our MR finding suggested that no genetic evidence of serum vitamin D levels was significantly associated with AF risk. Further researches are necessary to explore the potential role and mechanisms of circulating serum vitamin D levels on AF.

## Introduction

Atrial fibrillation (AF) is a common arrhythmia contributing to substantial social and medical burdens with significant health and socioeconomic impact (1). The Global Burden of Disease project estimated a worldwide prevalence of AF in about 46.3 million individuals in 2016 (2). The prevalence of AF is estimated to rise to 16 million by 2050 in the United States and 14 million by 2060 in the European Union (3). AF is associated with high healthcare system utilization, low quality of life, and increased risk for hospitalization, heart failure, stroke, and death (4).

Vitamin D is an essential fat-soluble vitamin that undergoes 2 hydroxylation steps to produce the active form. The first of these produces 25-hydroxyvitamin D, which can be measured to determine vitamin D status (5). Vitamin D deficiency has become a pandemic health problem in the world (6). In recent decades, the focus has been on vitamin D deficiency and nonskeletal diseases risks, including various cardiovascular diseases (7, 8). However, unlike for the skeletal disease, the association between vitamin D deficiency or 25-hydroxyvitamin D levels and AF risks has been inconclusive. Two dose-response meta-analyses (9, 10) indicated that circulating vitamin D deficiency was associated with an increased risk of AF in the general population, which were not consistent with another meta-analysis of randomized controlled trials (11). Conclusions about causality cannot be drawn merely based on the presence of an association in an observational design, which was retrospective or cross-sectional in design with limited sample sizes and confounders.

To investigate the causal association between circulating vitamin D and AF risks is challenging due to the reverse causation and confounding. Mendelian randomization (MR) has emerged as a powerful method for identifying the causation between risk factors and diseases using genetic variants as instrument variables (IVs) (12). MR analysis can largely overcome the confounders with random assignment of an individual's genetic variants at conception. Moreover, the risk of reverse causation could also be minimized since the presence of a disease could not affect individuals' genotypes (13).

In our study, we applied a two-sample MR analysis to identify the potential causal association between circulating serum vitamin D levels (including serum vitamin D and its metabolite, 25-hydroxyvitamin D) and risk of AF using the summary statistics from the publicly available genome-wide association studies (GWAS) data.

## Methods

### Data Resources and Study Design

We searched GWAS to extract leading single-nucleotide polymorphisms (SNPs) as genetic instrumental variables. Summary statistic data for vitamin D levels were derived from a meta-analyzed GWAS for 35 biomarkers in the UK Biobank (UKB) in 304,818 participants of White British European ancestry (14). UK Biobank is a prospective cohort which recruited more than 500,000 men and women aged 40–96 years between 2006 and 2010, and their health is being followed on a long-term (15). Summary statistic data for 25-hydroxyvitamin D was drawn from the most recent GWAS on serum 25-hydroxyvitamin D from the SUNLIGHT consortium with 79,366 European-ancestry participants including 31 studies (16). This study identified 142 independent risk variants at 111 loci and prioritized 151 functional candidate genes likely to be involved in atrial fibrillation (16). Data for AF was obtained from the latest meta-analysis of GWASs for AF with six independent cohorts (The Nord-Trøndelag Health Study, Michigan Genomics Initiative, DECODE, UK Biobank, DiscovEHR Collaboration Cohort, and AF Gen Consortium) with more than 1,000,000 subjects of European ancestry, including 60,620 cases with AF and 970,216 controls (17). The details are presented in Table 1.

**Table 1:** Details of studies included and predictive strength of IVs in Mendelian randomization analyses (two-sided α = 0.05).

**Exposures/outcomes**	**Consortium**	**Ethnicity**	**Sample sizes**	**Model**	**R-squared % (of variance in Exposure)**	**F-statistic (total)**
Vitamin D	UK Biobank	European	304,818	Model 1	0.546	31.578
				Model 2	0.521	33.279
25-hydroxyvitamin D	SUNLIGHT	European	79,366	Model 1	1.095	146.428
				Model 2	1.059	169.767
Atrial fibrillation	HUNT, DECODE, MGI, DiscovEHR, UK Biobank, and AFGen Consortium	European	1,030,836	NA	NA	NA

*AF, atrial fibrillation; HUNT, The Nord-Trøndelag Health Study; DECODE, DiscovEHR, Collaborative analysis of Diagnostic criteria in Europe study; MGI, Michigan Genomics Initiative; AF Gen, Atrial Fibrillation Genetics. Model 1, SNPs were not extracted which were associated with any potential confounders of AF; Model 2, SNPs were extracted which were associated with any potential confounders of AF*.

**Figure 1:**
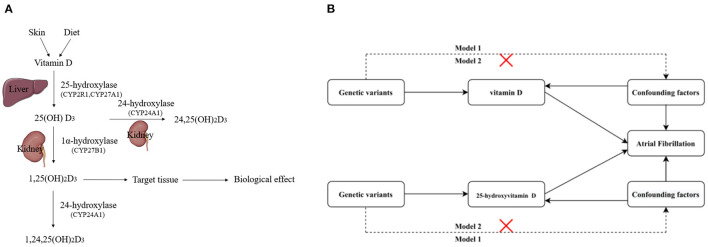
**(A)** Process of vitamin D metabolism. **(B)** Schematic overview of the present study design. Model 1, SNPs were not extracted which were associated with any potential confounders of AF; Model 2, SNPs were extracted which were associated with any potential confounders of AF. 25(OH)D_3_, 25-hydroxyvitamin D; 24,25(OH)_2_D_3_, 24,25-Dihydroxyvitamin D3; 1,25(OH)_2_D_3_, 1,25-Dihydroxyvitamin D3; 1,24,25(OH)_2_D_3_, 1,24,25-Dihydroxyvitamin D3.

We designed a two-sample Mendelian randomization analysis to estimate the causal effects of circulating serum vitamin D and 25-hydroxyvitamin D levels (recommended biomarker for vitamin D levels, Figure 1A) on AF risks with two models (Figure 1B). Model 2 was performed by extracting SNPs that were associated with any potential confounders on AF risks, while Model 1 was not.

### Selection of Genetic Instrumental Variables

All genetic variants reaching genome-wide significance (*p* < 5 × 10^−8^) were selected as instruments for the MR analysis. The corresponding linkage disequilibrium was tested to confirm if there were any SNPs in linkage disequilibrium and whether the SNPs were independent by pruning SNPs within a 10,000 kb window with an *r*^2^ < 0.001 threshold (18). Then, the SNPs were extracted that were associated with any potential confounders of the outcomes. In this study, blood pressure, blood glucose, BMI, chronic nephropathy, coronary artery disease (CAD), and C-reactive protein were identified as confounding factors when AF was identified as the outcome (http://www.phenoscanner.medschl.cam.ac.uk/) (19). SNP harmonization was conducted to correct the orientation of the alleles. Finally, we used 62 SNPs and 56 SNPs (3 SNPs were associated with BMI: rs56675301, rs35635959, and rs1229984 and 3 SNPs were associated with CAD: rs2207132, rs2229742, and rs2539986) as instrument variables for Vitamin D levels in model 1 and model 2, 6 SNPs and 5 SNPs (1 SNP was associated with white blood cell: rs10745742) for 25-hydroxyvitamin D levels in model 1 and model 2, respectively (Supplementary Tables 1–4). F statistics for every instrument-exposure effect ranged from 31.678 to 169.767, demonstrating the small possibility of weak instrumental variable bias (Table 1). In another directional MR, we used 13 SNPs and 42 SNPs for AF on vitamin D and 25-hydroxyvitamin D levels, respectively, and no SNP was associated with confounding factors when AF was identified as the exposure.

### Statistical Analysis

T To obtain an MR estimate, an inverse variance weighted (IVW) meta-analysis of each Wald Ratio (20) was performed. When there was no evidence of directional pleiotropy (*P* for MR-Egger intercept > 0.05) among the selected IVs, the IVW method was considered with the most reliability (21).

Complementary analyses using the weighted median method (22), maximum likelihood (23) and MR-egger method (22), and MR Robust adjusted profile score (MR.RAPS) were utilized as supplements to IVW. The weighted median analysis can generate consistent estimates if at least 50% of the weight in the analysis comes from valid instrumental variables (24). Cochran's Q test was applied to assess heterogeneity between individual genetic variants estimates. If the *p*-value of Cochran's Q test was < 0.05, the final results of MR were referred to a multiplicative random-effects model of IVW; otherwise, a fixed-effects model was used (25). To examine whether there was a violation of the main MR assumptions due to directional pleiotropy, the MR-Egger test for directional pleiotropy was performed (22), where the intercept estimates the average pleiotropic effect across the genetic variants and can be a useful indicator of whether directional horizontal pleiotropy is driving the results of an MR analysis (26). The possible directional pleiotropy was were also examined by observing asymmetry in thefunnel plots to gauge the reliability of the current MR analyses. Finally, MR-PRESSO was performed to support the results by IVW method, which detects and corrects the effects from outliers, yielding causal estimates that were robust to heterogeneity (27). The leave-one-out sensitivity analyses were implemented by removing a single SNP each time to assess whether the variant was driving the association between the exposure and the outcome variable. To improve the visualization of the IVW and MR-Egger estimates, we performed IVW radial variants and MR-Egger radial variants models, which were similar to the conventional IVW and MR-Egger regression models, but regressed the product of the Wald Ratio estimate and the square root of the weighting for each genetic variant upon the square root of the genetic variants weighting (28). *R*-squared was calculated to estimate the proportion of variance in outcomes, and F-statistic value was calculated to predict the strength of IVs.

A two-sided *P*-value of < 0.05 was considered suggestive for significance. All analyses were performed using the package “Two-Sample-MR” (version 0.5.6), “MR-PRESSO” (version 1.0), and “Radial MR” (version 1.0) in R (version 4.0.5).

## Results

### Association of Serum Vitamin D Levels With AF Risks

**Figure 2:**
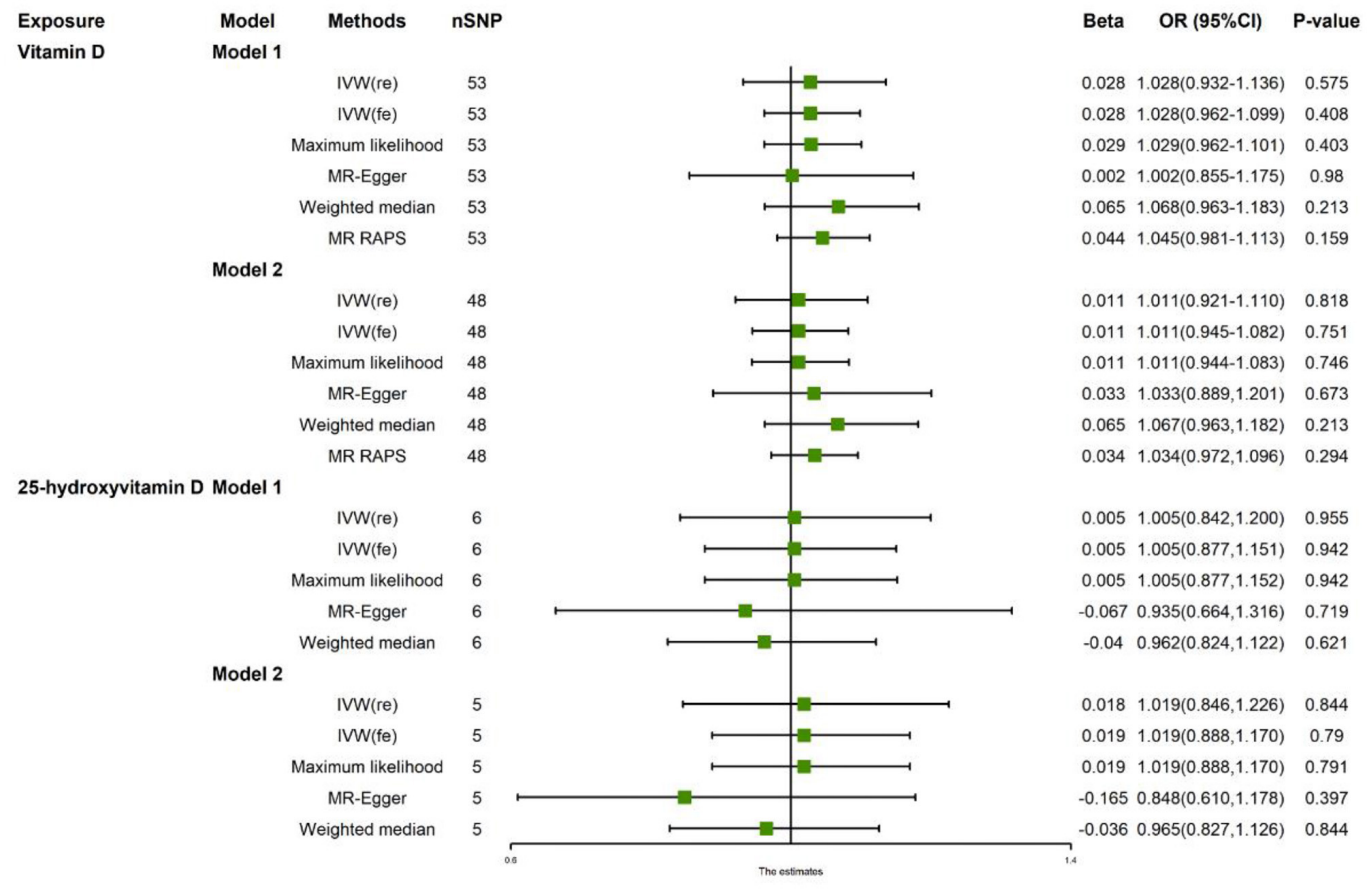
Associations of vitamin D and 25-hydroxyvitamin D levels with AF in two-sample Mendelian randomization analysis. SNPs, single nucleotide polymorphisms; IVW, inverse variance weighted; OR, odds ratio; RAPS, robust adjusted profile score.

Figure 2 reported the MR estimated for vitamin D levels on AF. In model 1, the fixed-model IVW estimate showed that genetically predicted vitamin D levels were not significantly associated with AF risks (*N* = 53 SNPs, OR: 1.028, 95% CI: 0.962–1.099, *p* = 0.408). After extracting 6 SNPs, the result was consistent (*N* = 48 SNPs, OR: 1.011, 95% CI: 0.945–1.082, *P* = 0.751). The association was consistent in complementary analyses using weighted-median method, maximum likelihood, MR-egger, and MR-RAPS method.

**Figure 3:**
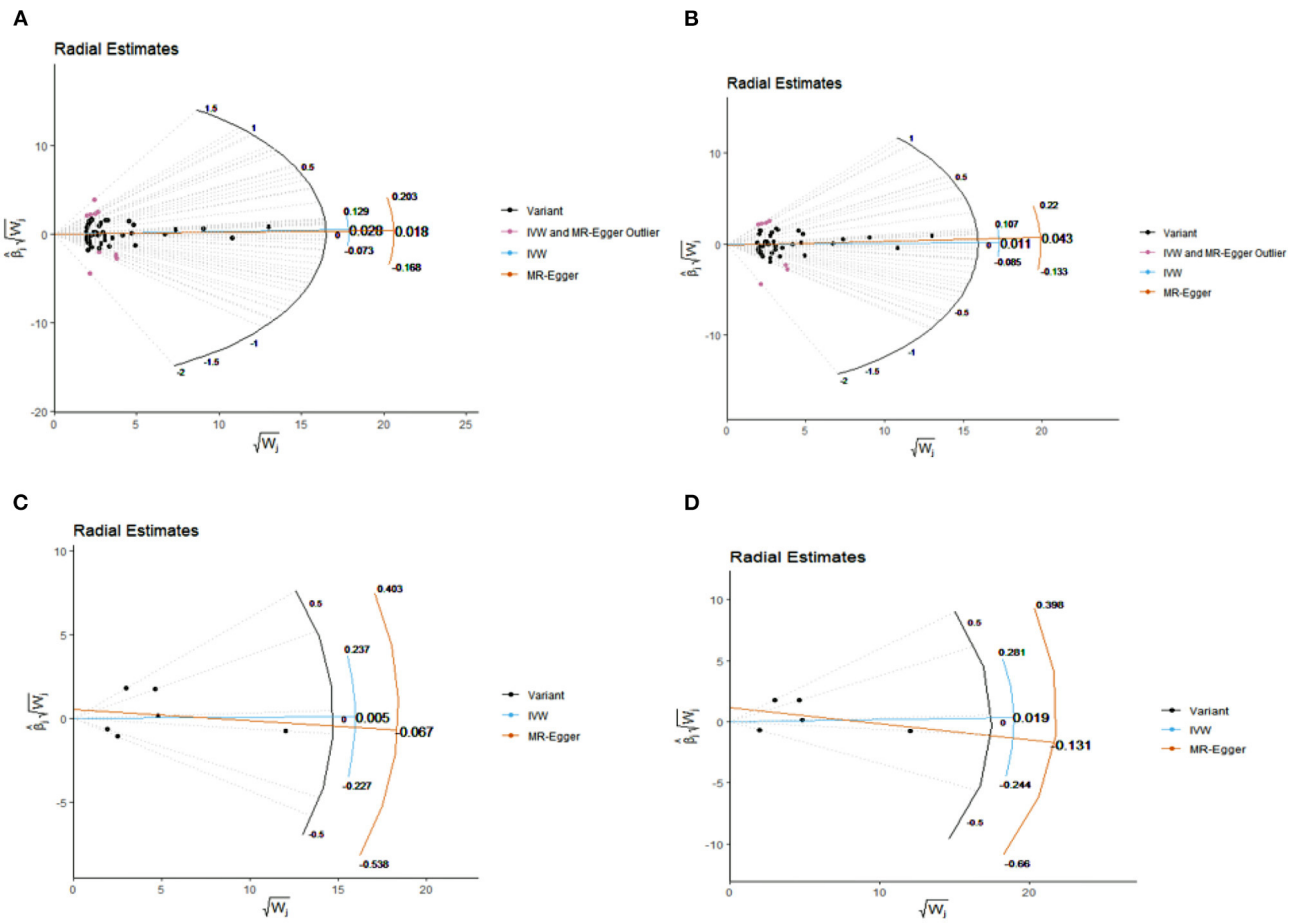
Radial plots to visualize individual outlier single nucleotide polymorphisms (SNPs) in the Mendelian randomization (MR) estimates for association between vitamin D with AF by model 1 **(A)** and model 2 **(B)** or association between 25-hydroxyvitamin D with AF by model 1 **(C)** and model 2 **(D)**. Black dots show valid SNPs and purple dots display invalid outlier SNPs. There is no significant outlier SNP in present plots C and D. IVW, indicates inverse-variance weighted.

There were potential heterogeneities but no directional pleiotropies for the analysis results (Supplementary Table 5). Radial plots showed there were outlines in Model 1 and Model 2 (Figures 3A,B). To ensure the robustness of our results, MR-PRESSO was also conducted with outliner correction which showed consistent results that vitamin D levels had no effect on the risk of AF (Table 2).

**Table 2:** MR-PRESSO for causal effect between vitamin D and AF.

**Exposure**	**Model**	**Raw estimates**				**Outlier corrected estimates**				**Distortion test**
		**nSNP**	**Beta**	**OR (95%CI)**	***P*-value**	**nSNP**	**Beta**	**OR (95%CI)**	***P*-value**	***P*-value**
Vitamin D	Model 1	53	0.039	1.040 (0.954,1.133)	0.368	50	0.05	1.051(0.978,1.130)	0.173	0.741
	Model 2	48	0.021	1.021 (0.942,1.107)	0.613	47	0.03	1.031(0.958,1.108)	0.422	0.812
25-hydroxyvitamin D	Model 1	6	0.005	1.005 (0.842,1.199)	0.958	6	NA	NA	NA	NA
	Model 2	5	0.019	1.019 (0.846,1.226)	0.853	5	NA	NA	NA	NA

*AF, atrial fibrillation; SNP, single-nucleotide polymorphisms; OR, odds ratio*.

The scatter plots and forest plots are displayed in Supplementary Figures 1A,B, 2A,B. The funnel plots were symmetrical (Supplementary Figures 3A,B), and the leave-one-out analysis revealed that no individual SNP was substantially driving the association between vitamin D and AF (Supplementary Figures 4A,B).

### Association of Serum 25-Hydroxyvitamin D Levels With AF Risks

In model 1, the random-model IVW estimate showed that genetically predicted 25-hydroxyvitamin D levels were not significantly associated with AF risks (*N* = 6 SNPs, OR: 1.005, 95% CI: 0.842–1.200, *P* = 0.955). After extracting 1 SNPs, the result was consistent (*N* = 5 SNPs, OR: 1.019, 95% CI: 0.846–1.226, *P* = 0.621). The association was consistent in complementary analyses by weighted median method, maximum likelihood, and MR-egger method, while MR-RAPS method was not applicable for limited SNPs. There were no potential heterogeneities and no directional pleiotropies for the analysis results (Supplementary Table 5). Radial plots showed there were no outlines both in model 1 and model 2 (Figures 3C,D). To ensure the robustness of our results, MR-PRESSO was also conducted with outliner correction, which showed similar results that vitamin D levels were not associated with the risk of AF (Table 2).

The scatter plots, forest plots, and funnel plots aredisplayed in Supplementary Figures 1C,D, 2C,D, 3C,D, and the leave-one-out analysis indicated that no individual SNP was substantially driving the association between them (Supplementary Figures 4C,D).

### Association of Serum AF With and Vitamin D and 25-Hydroxyvitamin D Levels

The IVW method estimate showed that genetically predicted AF was not significantly associated with vitamin D and 25-hydroxyvitamin D levels risks (*N* = 13 SNPs, OR: 1.032, 95% CI: 0.977–1.075, *p* = 0.057; *N* = 42 SNPs, OR: 0.997, 95% CI: 0.989–1.006, *P* = 0.527, Supplementary Table 5). The association was consistent in MR-PRESSO (Supplementary Table 8). The scatter plots, forest plots and funnel plots were displayed in Supplementary Figures 1E,F, 2E,F, 3E,F, and the leave-one-out analysis indicated that no individual SNP was substantially driving the association between them (Supplementary Figures 4E,F).

## Discussion

In this two-sample MR study, we found no significant causal relationship between serum vitamin D levels and AF risks.

There is consistent evidence to show that low serum 25-hydroxyvitamin D levels are associated with increased risk of cardiovascular diseases, including hypertension, coronary artery disease, ischemic heart disease, and stroke (7, 29–32). However, the causal relationship between vitamin D and AF is inconclusive. Previous retrospective studies investigated the positive relationship between vitamin D and AF risks. For example, Chen et al. (33) found that the serum 25(OH)D level was significantly lower in the AF group than in the nonAF group. However, this trial was not randomized, prospective, and blinded, and low vitamin D levels could be presented in those without AF, so that a mechanistic cause of low vitamin D was not proven. Other two studies (34, 35) also showed the preventive role of vitamin D in patients with AF. These two studies enrolled AF patient with hypertension and chronic heart failure, which are risk factors of AF, respectively. It seemed that positive results observed in these studies were amplified by confounding factors, including the other cardiovascular diseases.

Several prospective cohort study and RCTs have been performed to investigate the cause–effect of vitamin D supplementation on AF. The Rotterdam Study (36), the Multi-Ethnic Study of Atherosclerosis (MESA) (37) and the Cardiovascular Health Study (CHS) (37) all showed vitamin D deficiency was not associated with the occurrence of AF. A latest meta-analysis suggested that vitamin D deficiency was modestly associated with the occurrence of AF on a pooled analysis of case–control studies, while there appeared to be no association on pooled analysis of cohort studies (10). The discrepancy among the findings of many observational studies is likely due to the residual confounding. Our results are in accordance with the most recent meta-analysis of randomized controlled trials, which showed that serum vitamin D might not to play a major role in the development of new-onset AF (11).

Different from the other CVDs, AF is a complex arrhythmia that could be the outcome of various pathophysiological processes (38). The pathophysiology of AF included the basic electrophysiological and structural changes within the left atrium, the genetics of AF, and wider systemic and metabolic perturbations (38, 39). At present, the association between serum vitamin-D levels and AF has several potential pathophysiological mechanisms. Firstly, 1,25[OH]D, the activated form of vitamin D, inhibits the renin–angiotensin–aldosterone system (RAAS) (40, 41). RAAS plays a role in both structural and electrical remodeling of the atrium, suppresses cardiac myocyte hypertrophy and reduces inflammation (42). It can be inferred vitamin D deficiency may impair the prevention of AF by inhibiting RAAS. Secondly, vitamin D was associated with an inflammatory milieu and could increase the synthesis of C-reactive protein (CRP) directly or indirectly, which was crucial for the pathogenesis of AF (43). However, studies have suggested that vitamin D deficiency may be a consequence, not a cause of inflammation (44). In a word, the potential mechanisms of vitamin D and AF are still not fully illuminated and in dispute.

Our analysis has several strengths. Firstly, data from a large genetic consortium for serum vitamin D (*n* = 304,818), 25-hydroxyvitamin D levels (*n* = 79, 366), and AF (*n* = 1,030,836) allowed to increase the statistical power to detect small effects in complex phenotypes (45). Secondly, MR study avoided the potential biases based on the three core assumptions (46). Thirdly, the genetic variants used as the IVs were located in different chromosomes, the potential gene–gene interaction might have little effect on the estimated value (47). Furthermore, the sensitivity analysis with different MR methods showed consistent effects, including the radial plots and MR-PRESSO process. All the results showed no significant causal effects of serum vitamin D levels on AF risks.

There are some limitations in our study. Firstly, there were some heterogeneities in the study. Due to the GWAS data, any potential factors related to health status, age, and sex might contribute to the heterogeneities. Secondly, our study could not rule out the effect of canalization (i.e., dilution of the gene-exposure association), and thus the estimate might be inflated (48). Thirdly, the directional pleiotropy cannot be excluded, which is almost completely mediated through other causal pathways. Fourthly, the association between vitamin D deficiency and different AF subtypes was not explored because of the limited data, especially paroxysmal AF. Fifthly, our datasets included the European populations which limited applicability of results to non-European populations. Finally, there are potential biases in our studies caused by overlapping use of UK Biobank data. More studies are needed to verify the applicability of these results in other populations and other ethnicities in the future.

## Conclusion

Our MR study did not find the association between circulating vitamin D levels and the AF risks. Further studies in different ethnicities are necessary to explore the potential role and mechanisms of circulating serum vitamin D levels on AF.

## Data Availability Statement

The original contributions presented in the study are included in the article/Supplementary Material, further inquiries can be directed to the corresponding author.

## Author Contributions

SY wrote the manuscript, performed quality assessment, and statistic analysis. LW designed the project and edited the manuscript. HZ helped revised the manuscript for language and checked the results. YS checked the results. All authors contributed to the article and approved the submitted version.

## Funding

This study was supported by the National Natural Science Foundation of China (81673259), Natural Science Foundation of Jiangsu Province, China (BK20161435), and Jiangsu Commission of Health (H2019079).

## Conflict of Interest

The authors declare that the research was conducted in the absence of any commercial or financial relationships that could be construed as a potential conflict of interest.

## Publisher's Note

All claims expressed in this article are solely those of the authors and do not necessarily represent those of their affiliated organizations, or those of the publisher, the editors and the reviewers. Any product that may be evaluated in this article, or claim that may be made by its manufacturer, is not guaranteed or endorsed by the publisher.

## Abbreviations

AF: atrial fibrillation
MR: Mendelian randomization
SNP: single nucleotide polymorphisms
GWAS: genome-wide association
IVW: inverse variance weighted
IVs: instrument variables
UKB: UK Biobank
MR-PRESSO: MR pleiotropy residual sum and outlier test.
