# Supplementary material for: Circulating Vitamin D Levels and the Risk of Atrial Fibrillation: A Two-Sample Mendelian Randomization Study
Source: Front Nutr. 2022 Mar 28;9:837207. doi: 10.3389/fnut.2022.837207 (PMC8996811; doi:10.3389/fnut.2022.837207)

Supplementary materials

Figure S1 Scatter plot of MR effect size for causal associations

A. Vitamin D levels and atrial fibrillation (Model 1)

B. Vitamin D levels and atrial fibrillation (Model 2)

C. 25-hydroxyvitamin D levels and atrial fibrillation (Model 1)

D. 25-hydroxyvitamin D levels and atrial fibrillation (Model 2)

E. Atrial fibrillation and Vitamin D levels

F. Atrial fibrillation and 25-hydroxyvitamin D levels

Figure S2 Forest plot of MR effect size using MR-Egger and IVW methods for causal

associations

A. Vitamin D levels and atrial fibrillation (Model 1)

B. Vitamin D levels and atrial fibrillation (Model 2)

C. 25-hydroxyvitamin D levels and atrial fibrillation (Model 1)

D. 25-hydroxyvitamin D levels and atrial fibrillation (Model 2)

E. Atrial fibrillation and Vitamin D levels

F. Atrial fibrillation and 25-hydroxyvitamin D levels

Figure S3 Funnel plot of causal associations

A. Vitamin D levels and atrial fibrillation (Model 1)

B. Vitamin D levels and atrial fibrillation (Model 2)

C. 25-hydroxyvitamin D levels and atrial fibrillation (Model 1)

D. 25-hydroxyvitamin D levels and atrial fibrillation (Model 2)

E. Atrial fibrillation and Vitamin D levels

F. Atrial fibrillation and 25-hydroxyvitamin D levels

Figure S4 Leave-one-out plot to assess if a single variant is driving the association

A. Vitamin D levels and atrial fibrillation (Model 1)

B. Vitamin D levels and atrial fibrillation (Model 2)

C. 25-hydroxyvitamin D levels and atrial fibrillation (Model 1)

D. 25-hydroxyvitamin D levels and atrial fibrillation (Model 2)

E. Atrial fibrillation and Vitamin D levels

F. Atrial fibrillation and 25-hydroxyvitamin D levels

Figure S1 Scatter plot of MR effect size for causal associations

A. Vitamin D levels and atrial fibrillation (Model 1)


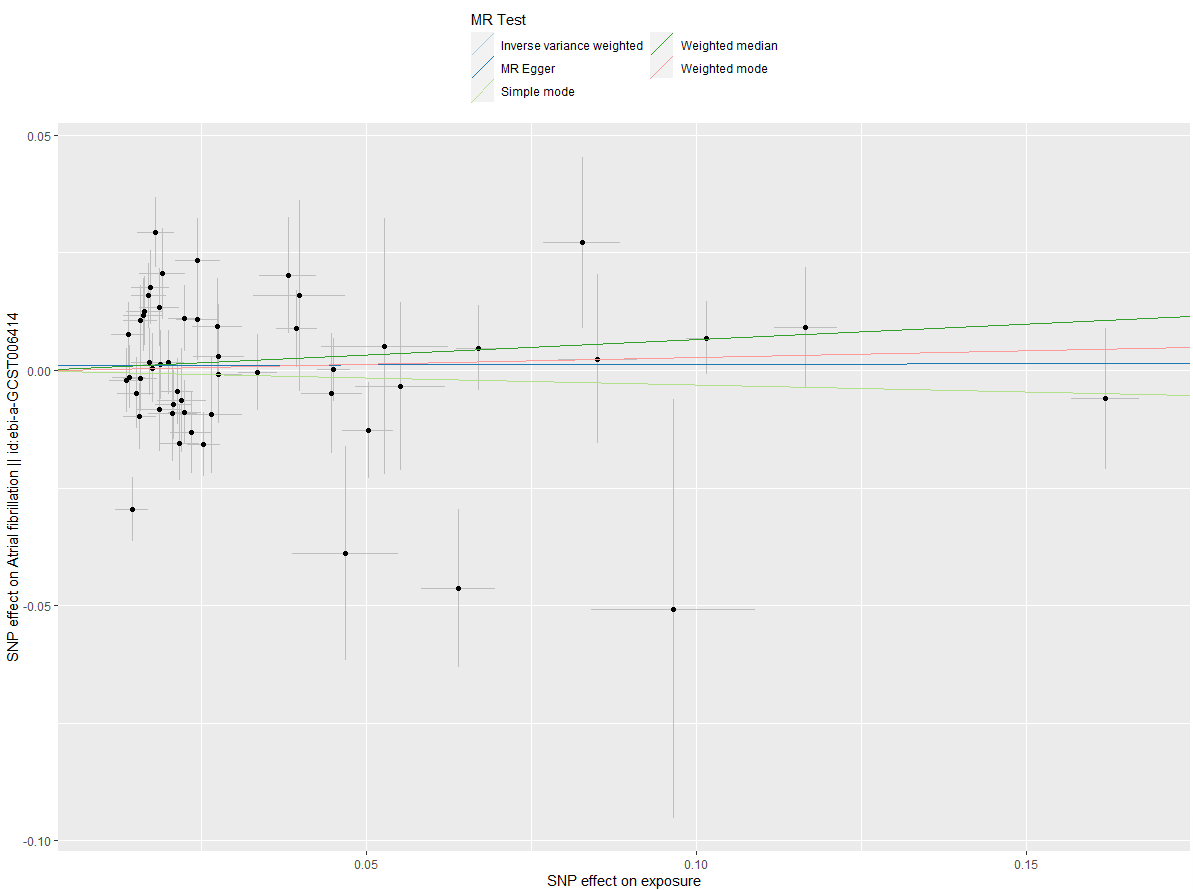


B. Vitamin D levels and atrial fibrillation (Model 2)


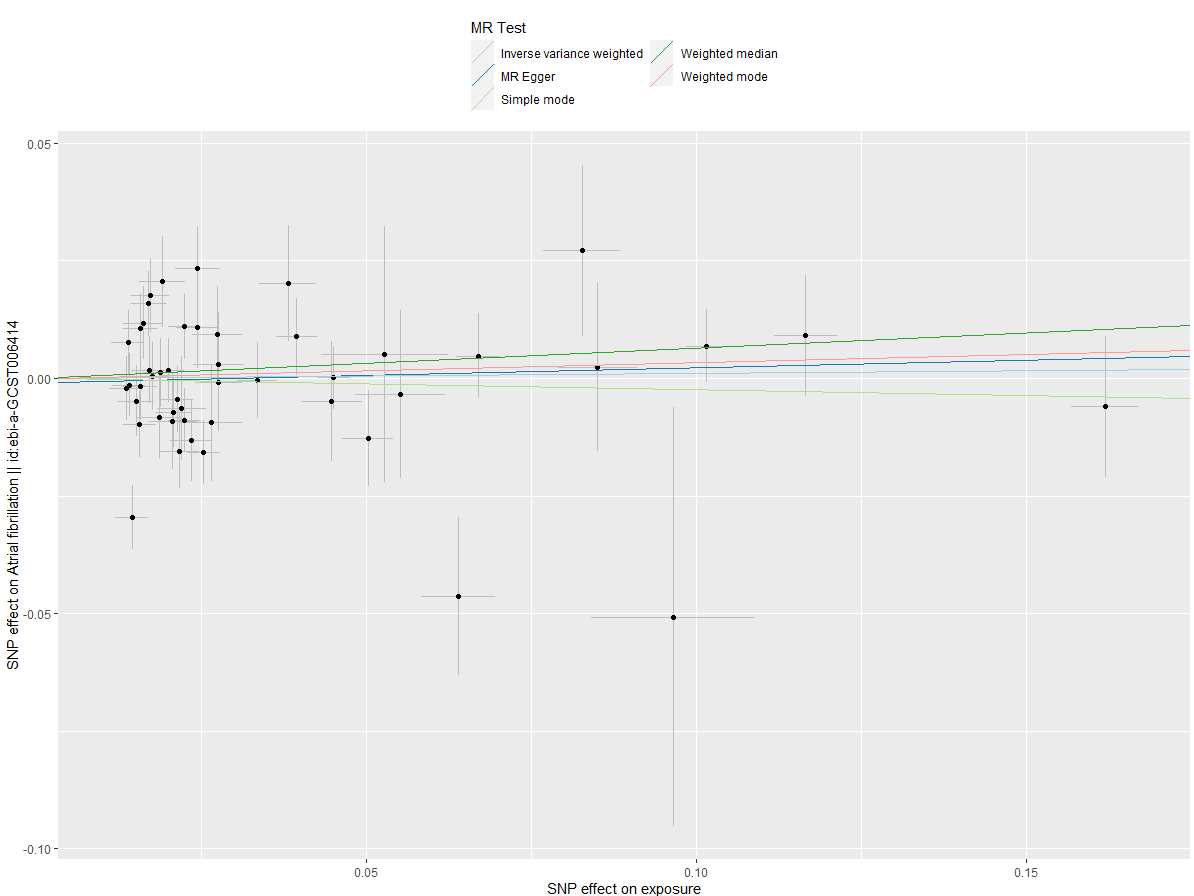


C. 25-hydroxyvitamin D levels and atrial fibrillation (Model 1)


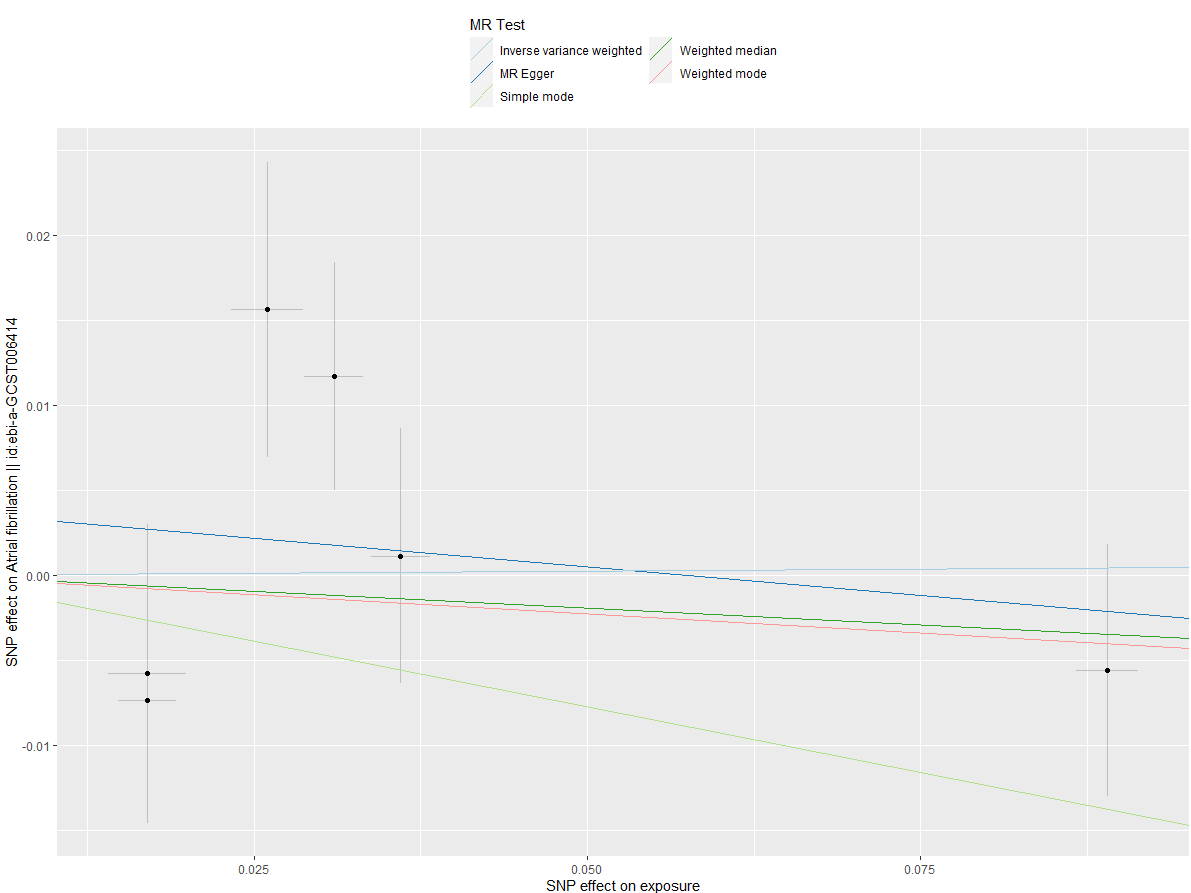


D. 25-hydroxyvitamin D levels and atrial fibrillation (Model 2)


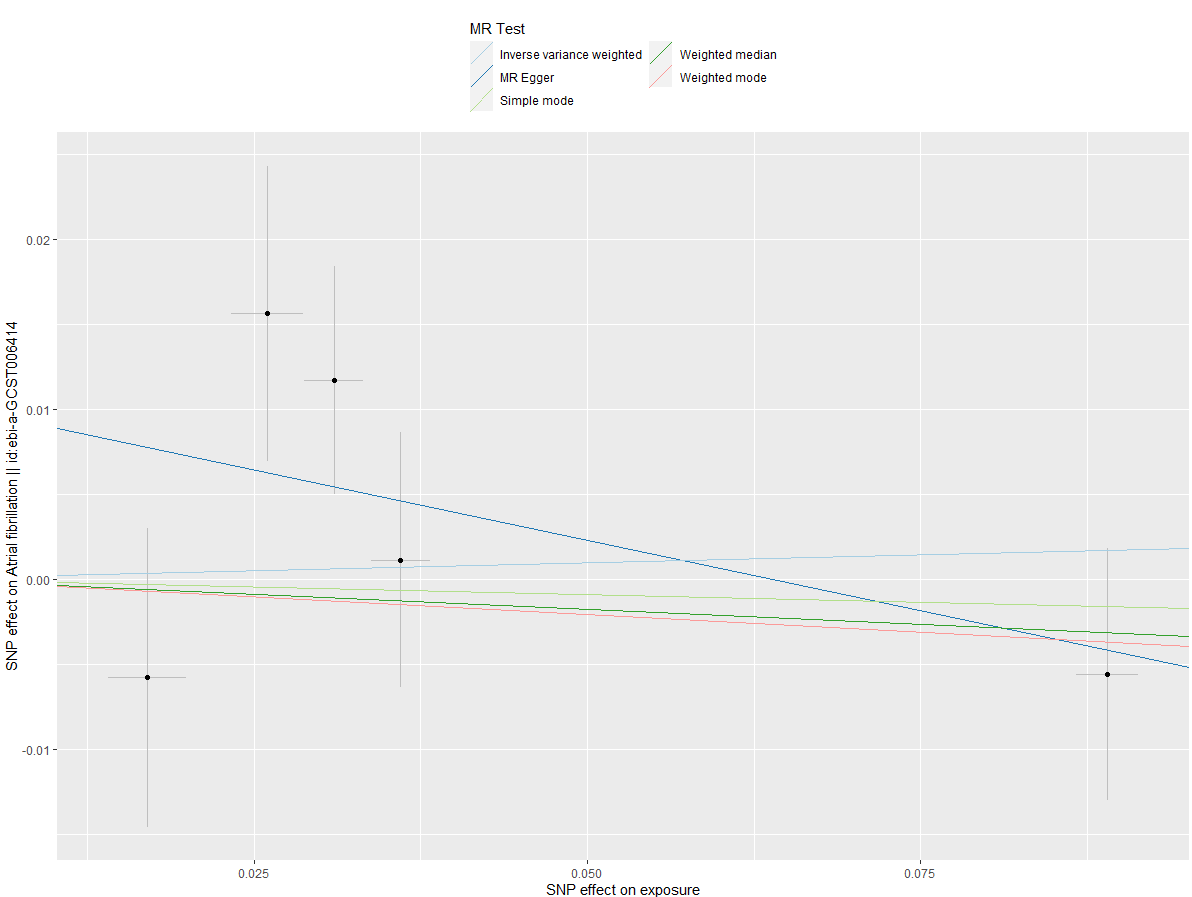


E. Atrial fibrillation and Vitamin D levels


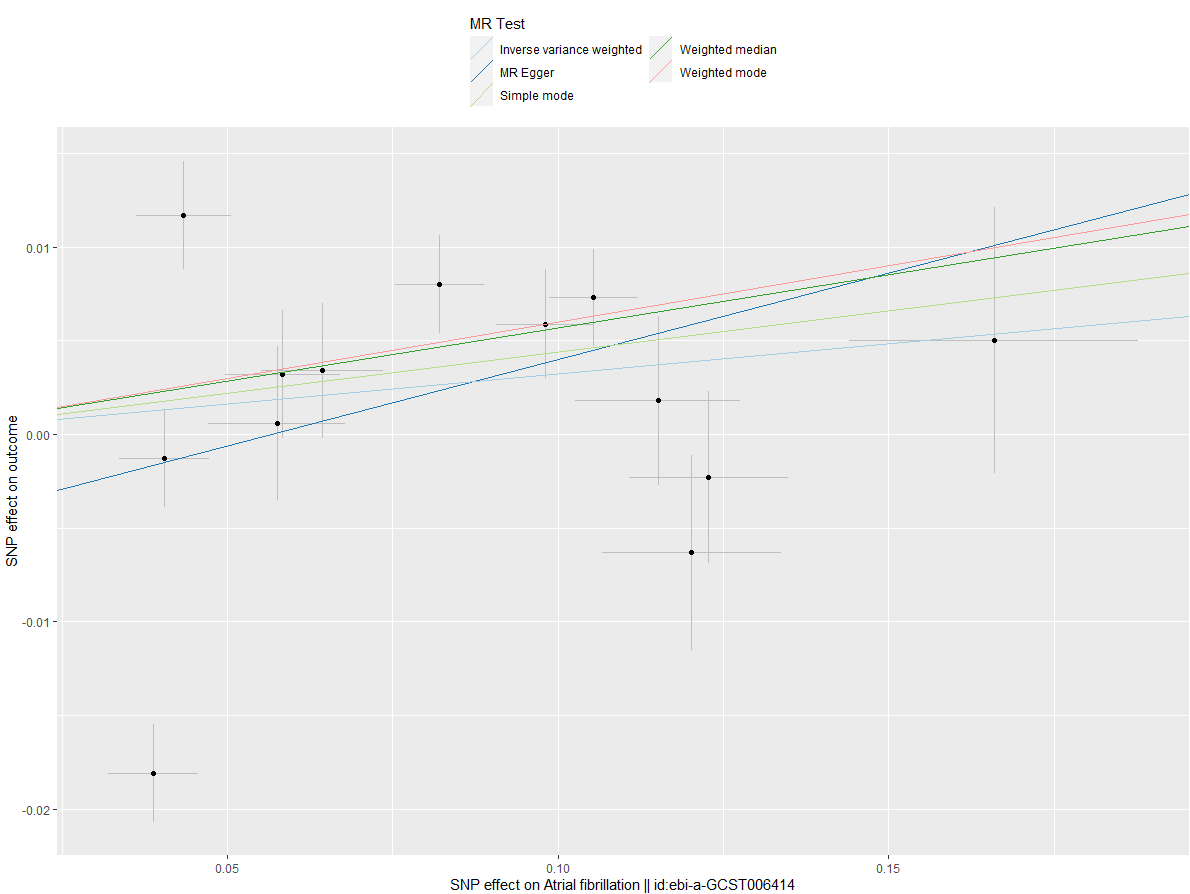


F. Atrial fibrillation and 25-hydroxyvitamin D levels


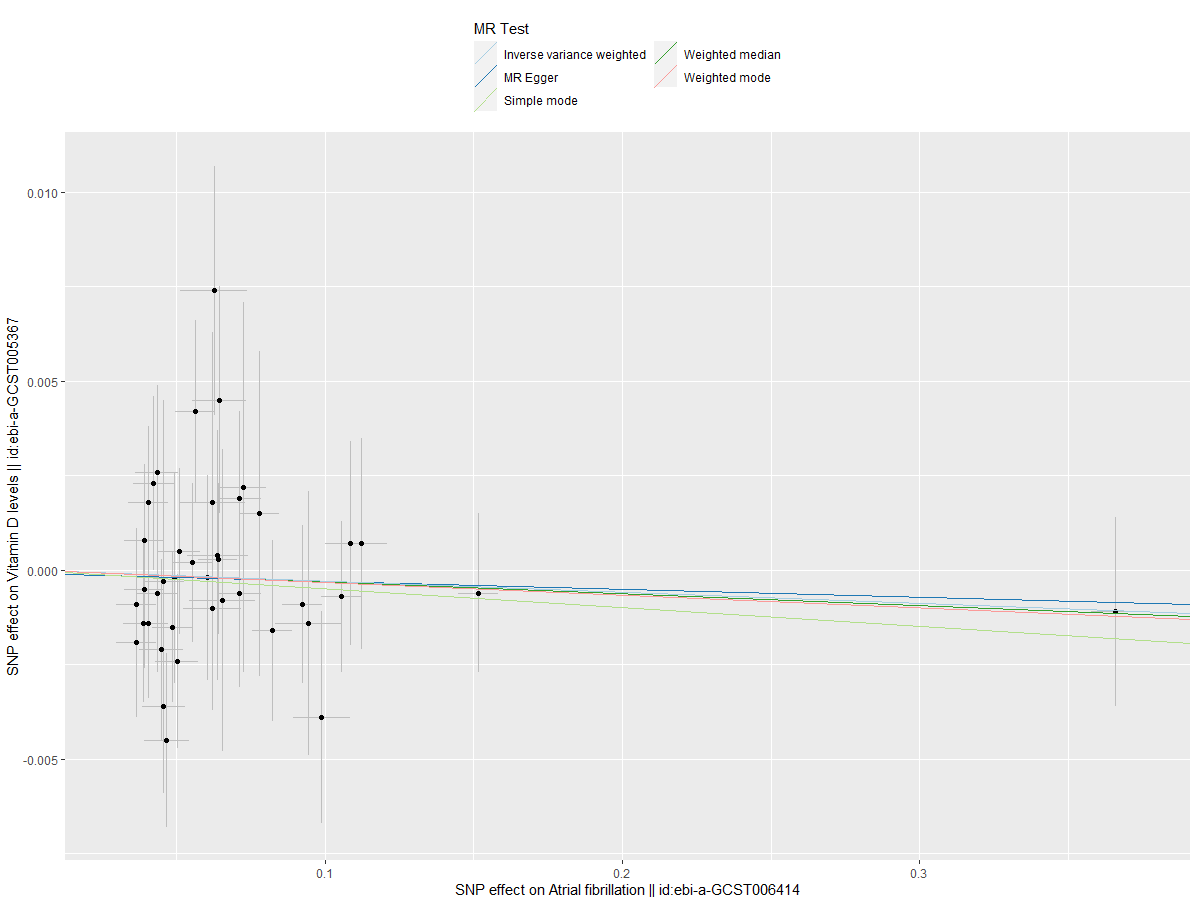


Figure S2 Forest plot of MR effect size using MR-Egger and IVW methods for causal

associations

A. Vitamin D levels and atrial fibrillation (Model 1)


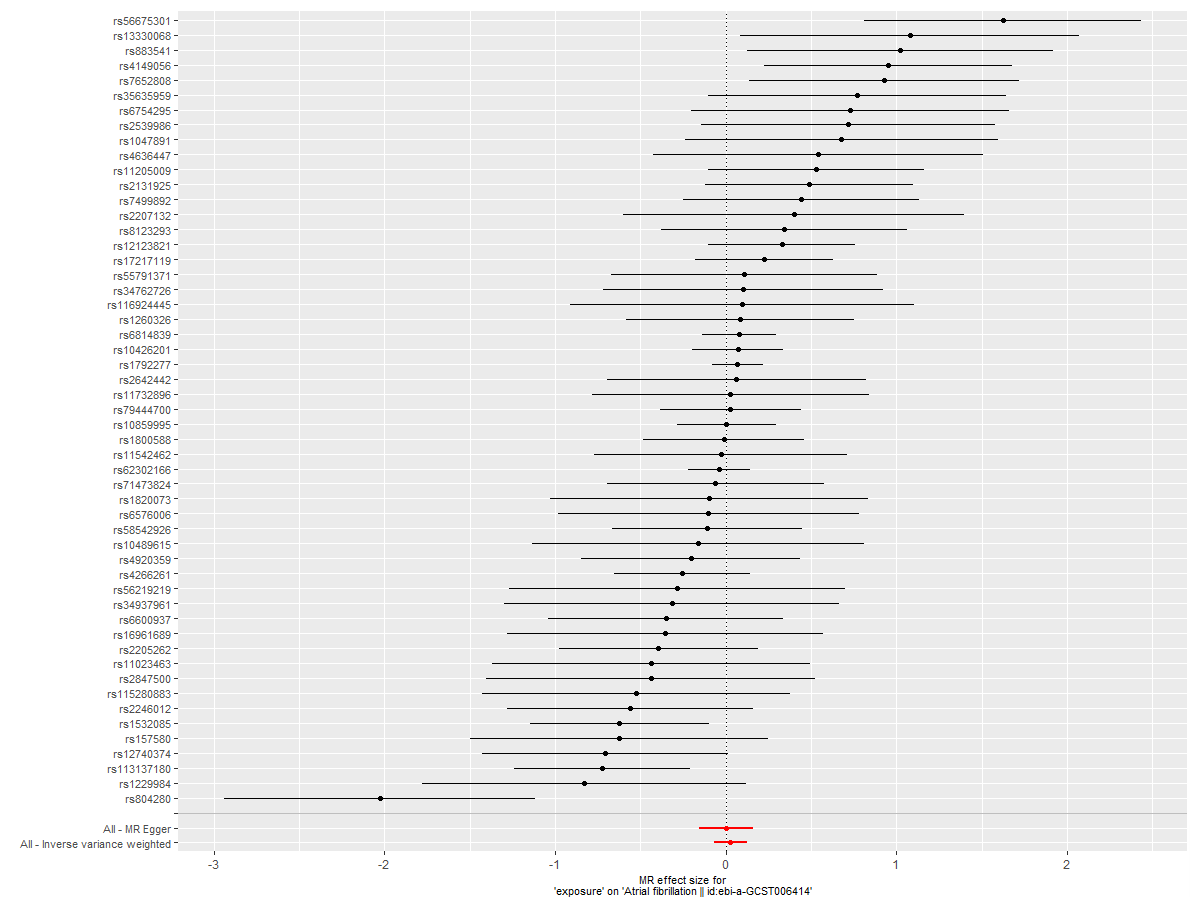


B. Vitamin D levels and atrial fibrillation (Model 2)


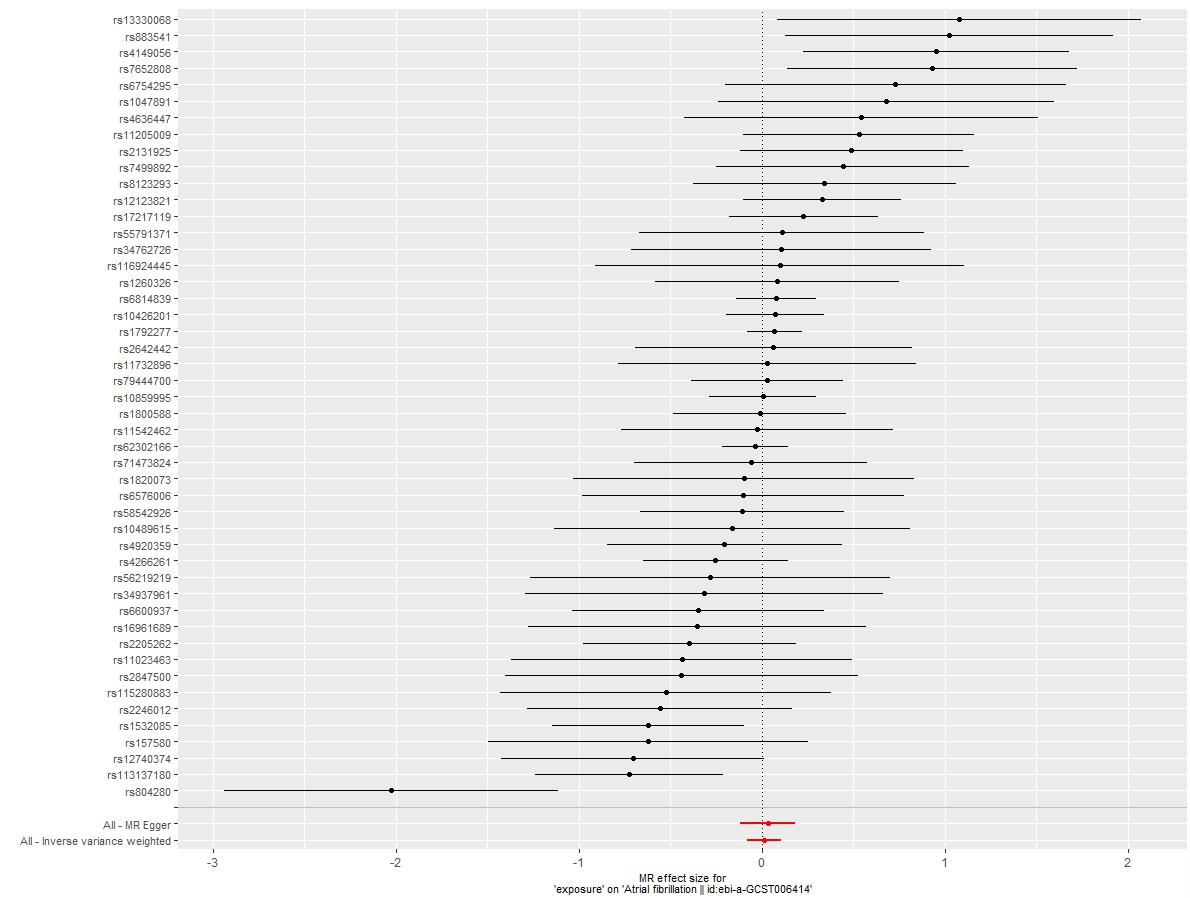


C. 25-hydroxyvitamin D levels and atrial fibrillation (Model 1)


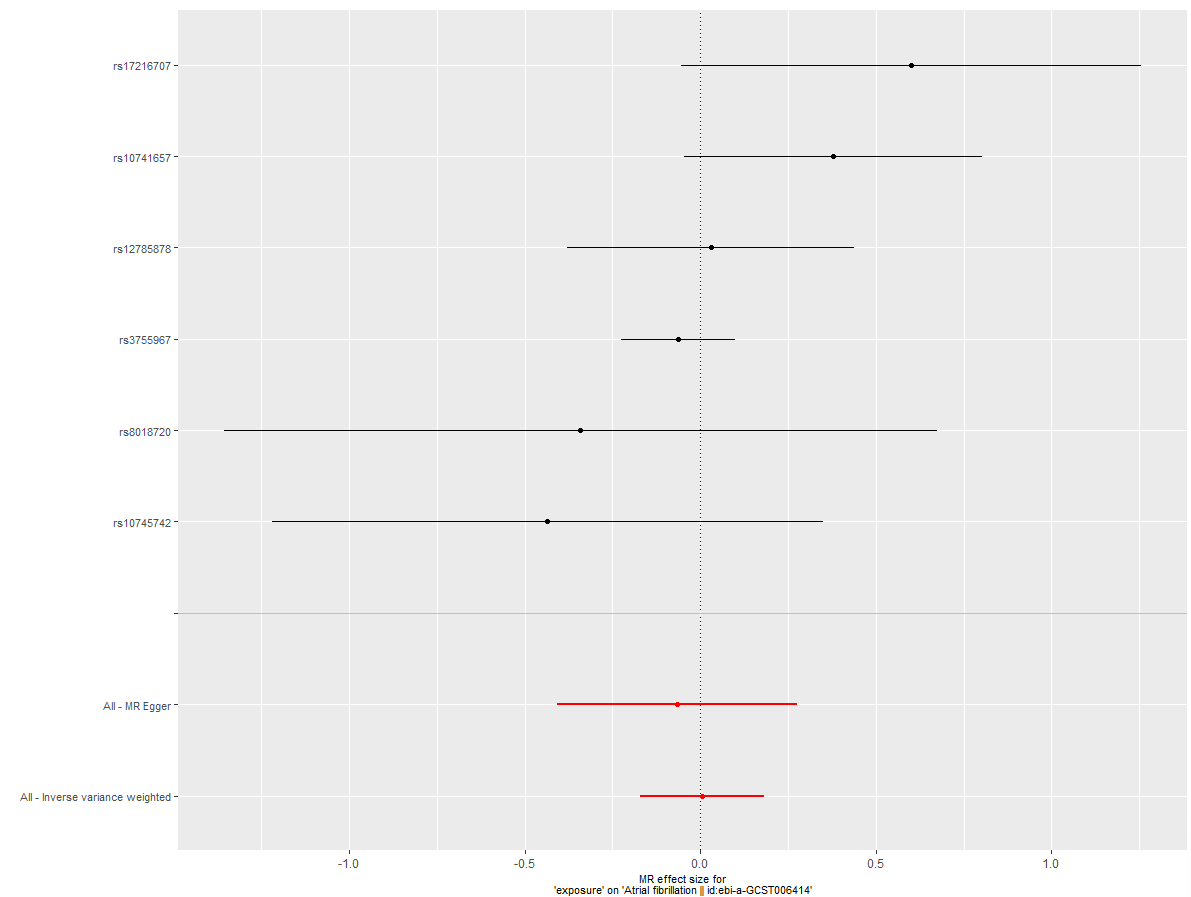


D. 25-hydroxyvitamin D levels and atrial fibrillation (Model 2)


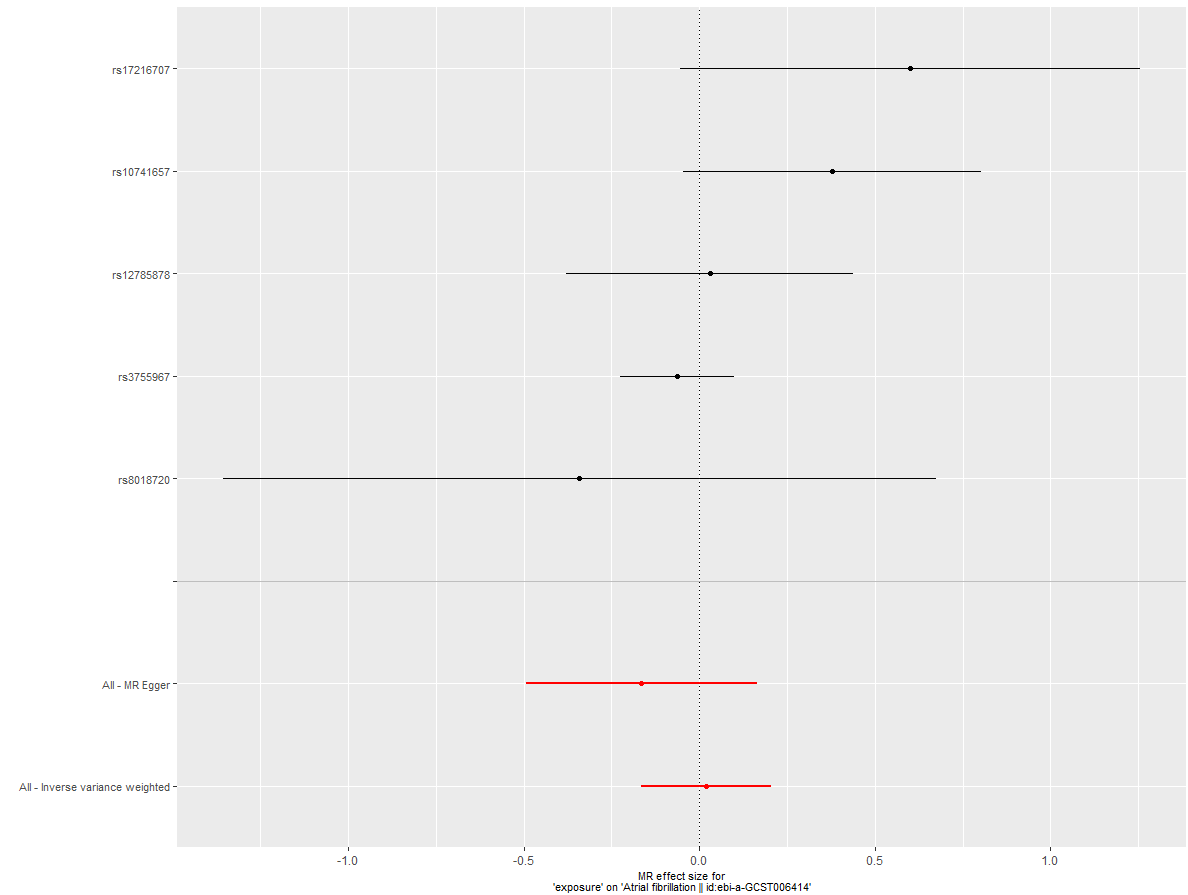


E. Atrial fibrillation and Vitamin D levels


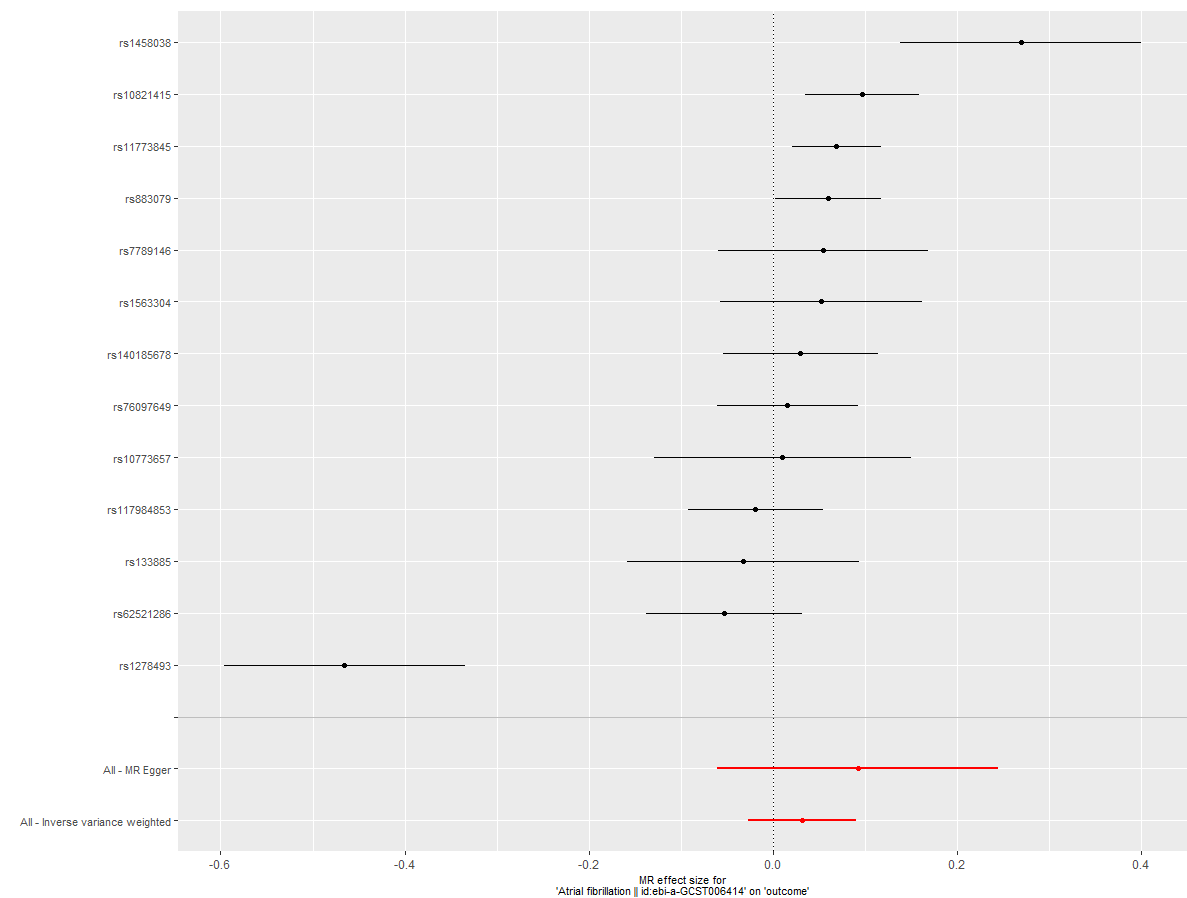


F. Atrial fibrillation and 25-hydroxyvitamin D levels


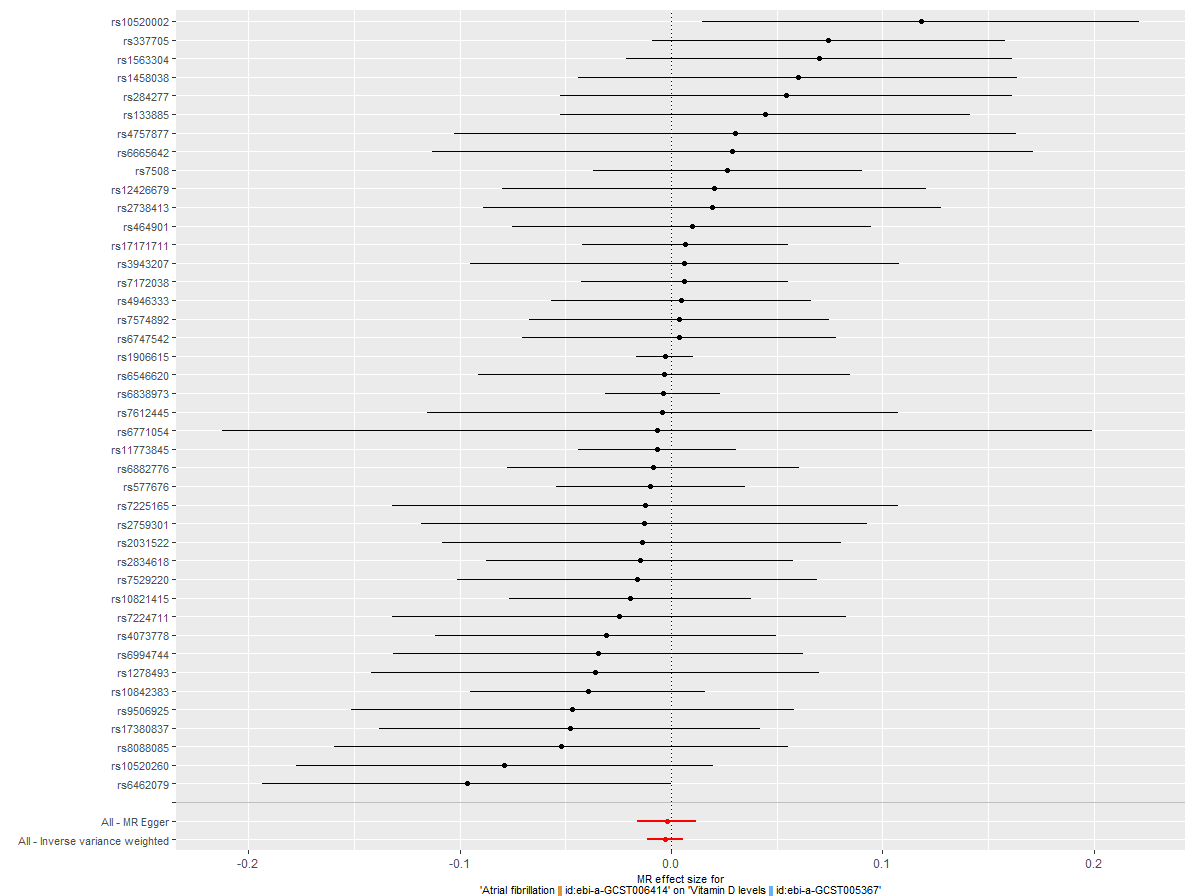


Figure S3 Funnel plot of causal associations

A. Vitamin D levels and atrial fibrillation (Model 1)


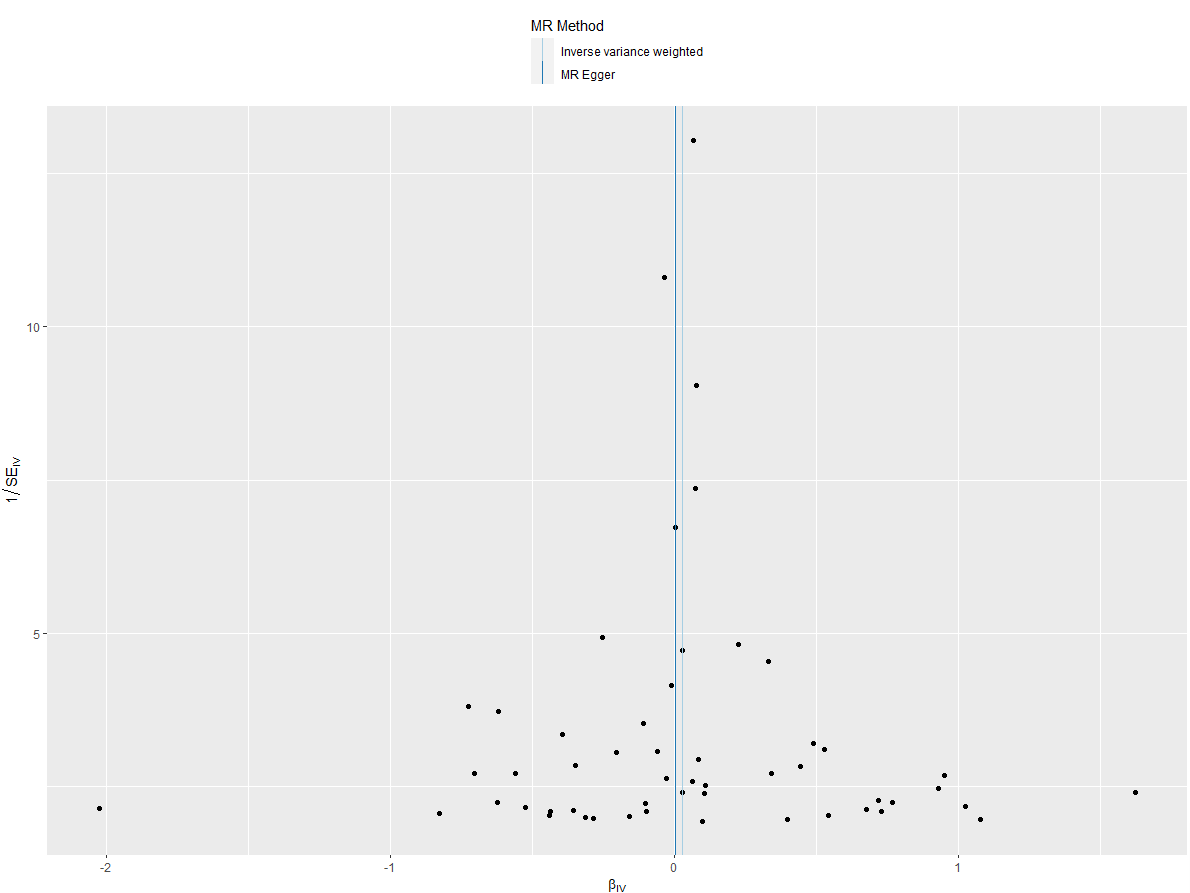


B. Vitamin D levels and atrial fibrillation (Model 2)


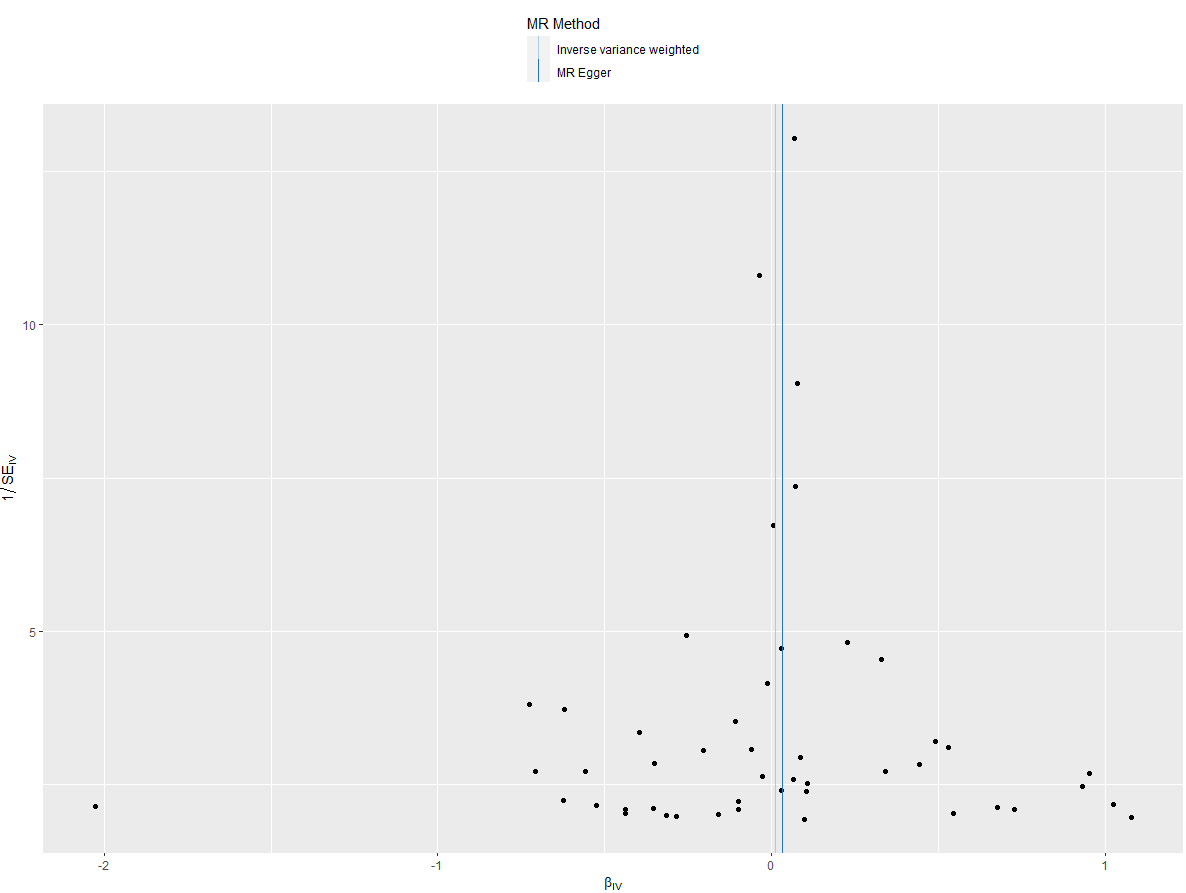


C. 25-hydroxyvitamin D levels and atrial fibrillation (Model 1)


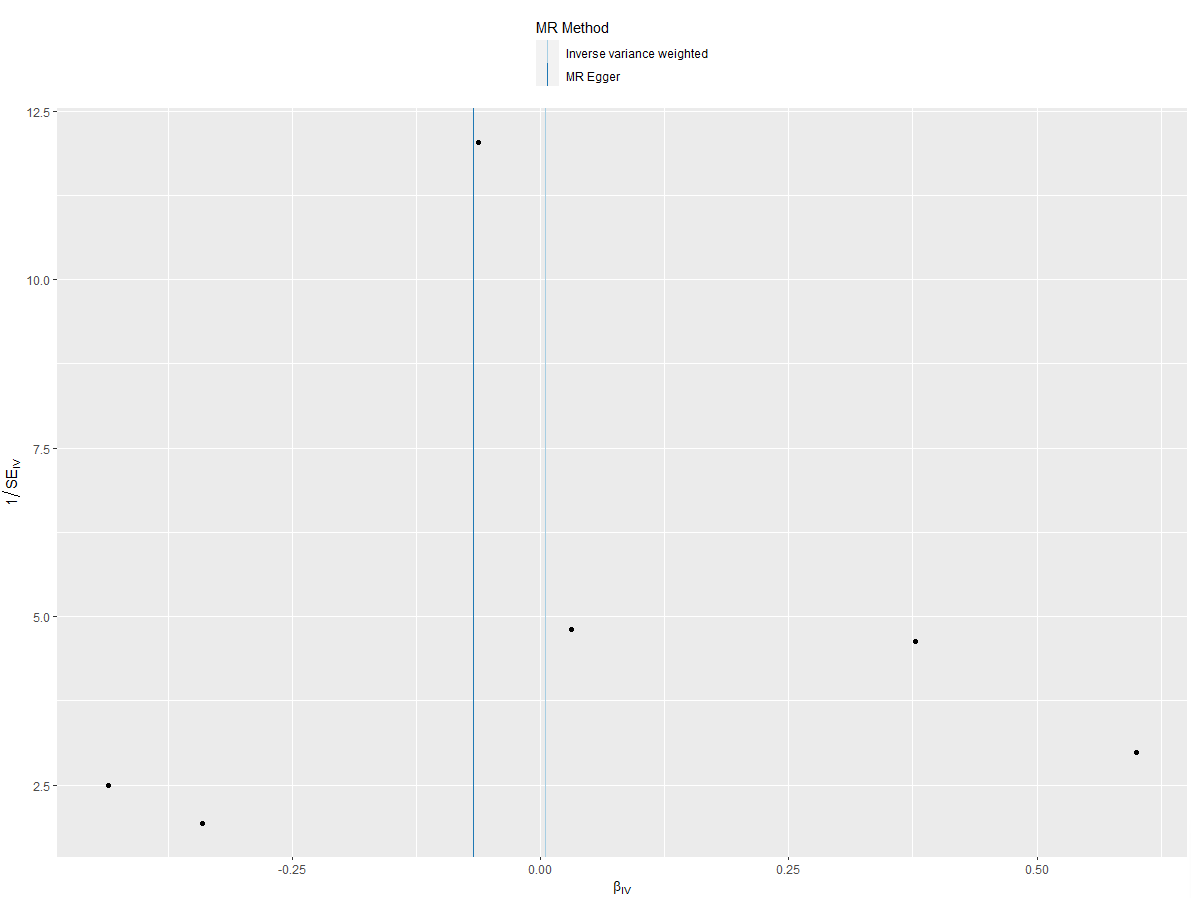


D. 25-hydroxyvitamin D levels and atrial fibrillation (Model 2)


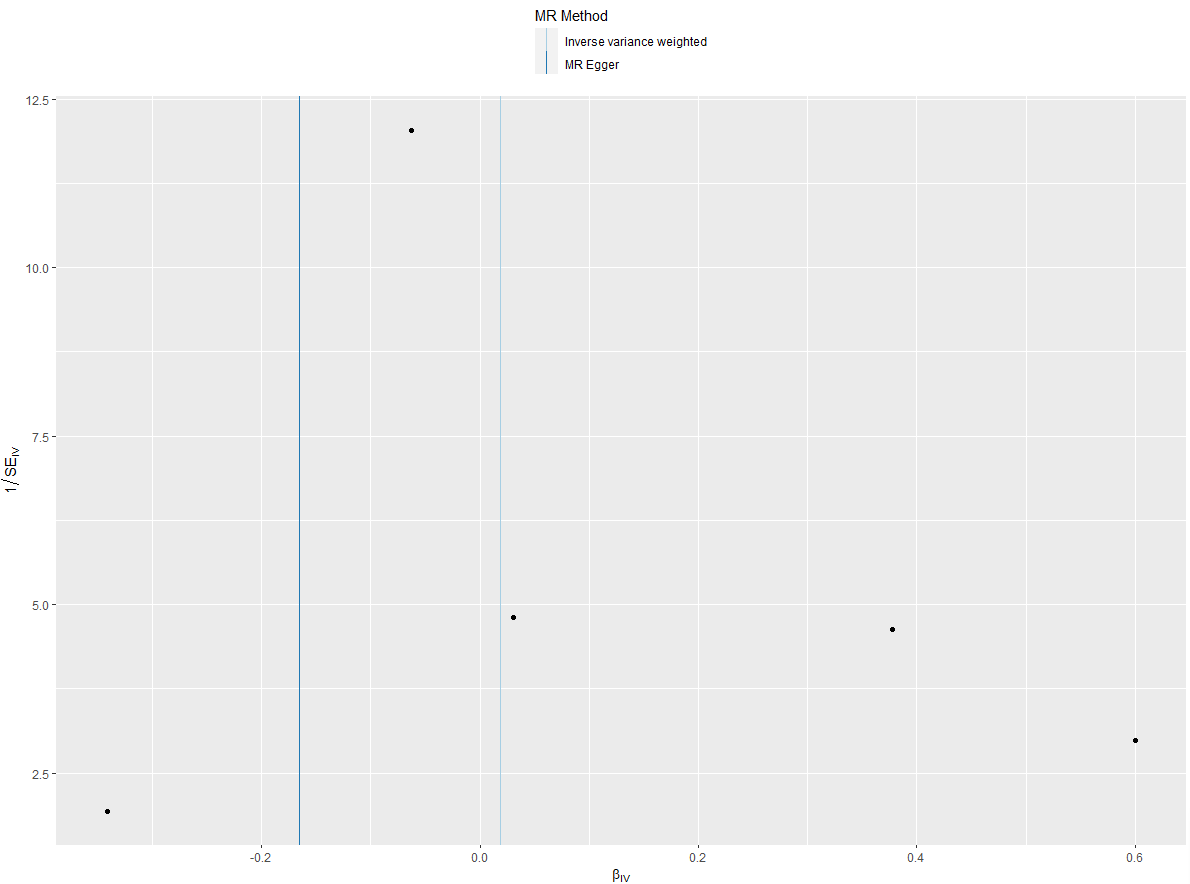


E. Atrial fibrillation and Vitamin D levels


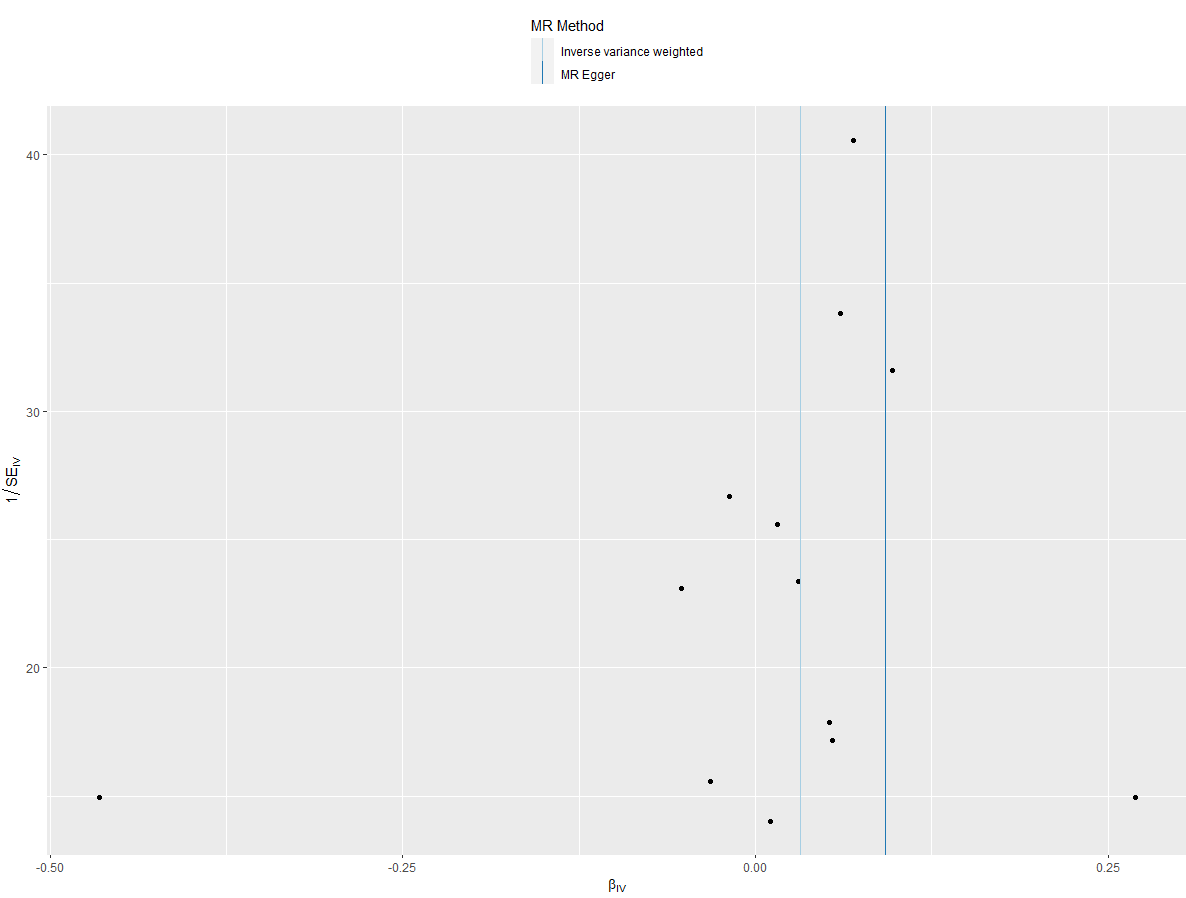


F. Atrial fibrillation and 25-hydroxyvitamin D levels


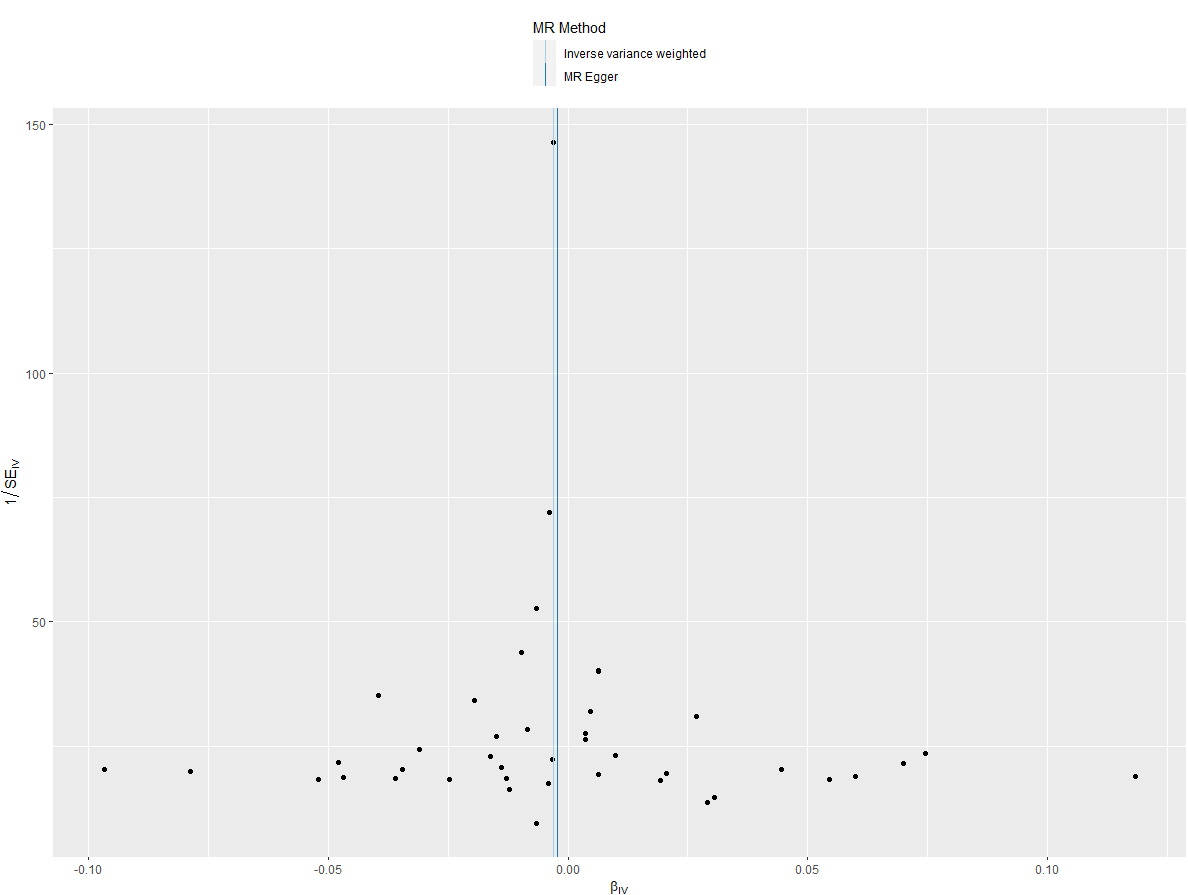


Figure S4 Leave-one-out plot to assess if a single variant is driving the association

A. Vitamin D levels and atrial fibrillation (Model 1)


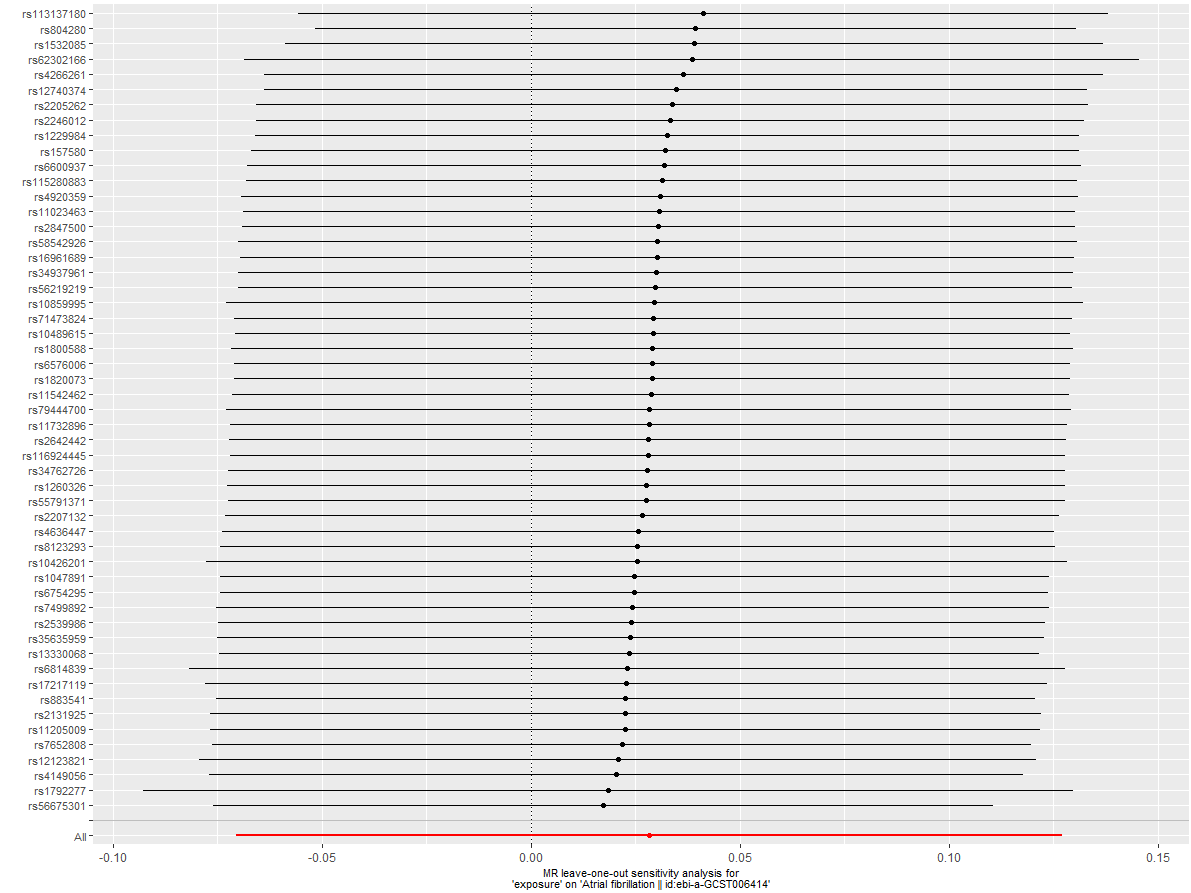


B. Vitamin D levels and atrial fibrillation (Model 2)


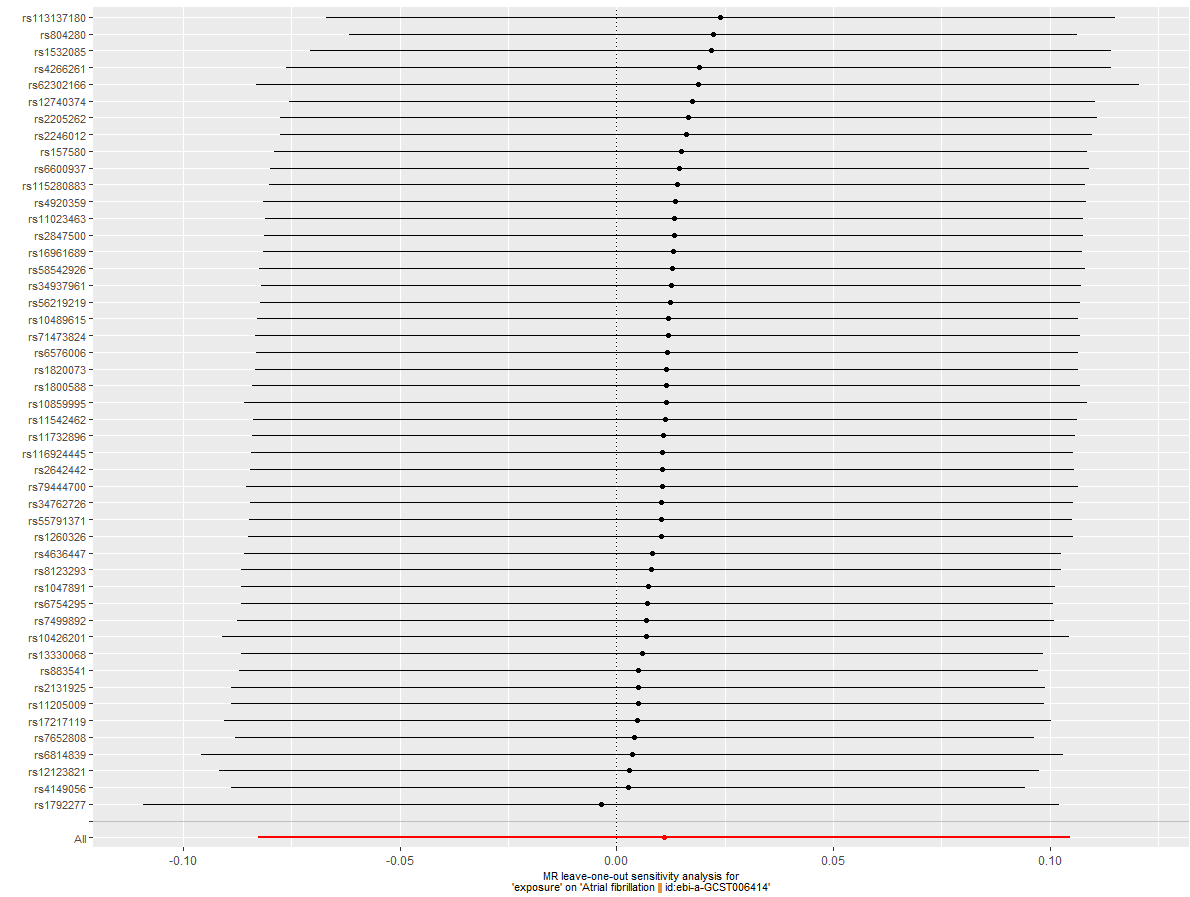


C. 25-hydroxyvitamin D levels and atrial fibrillation (Model 1)


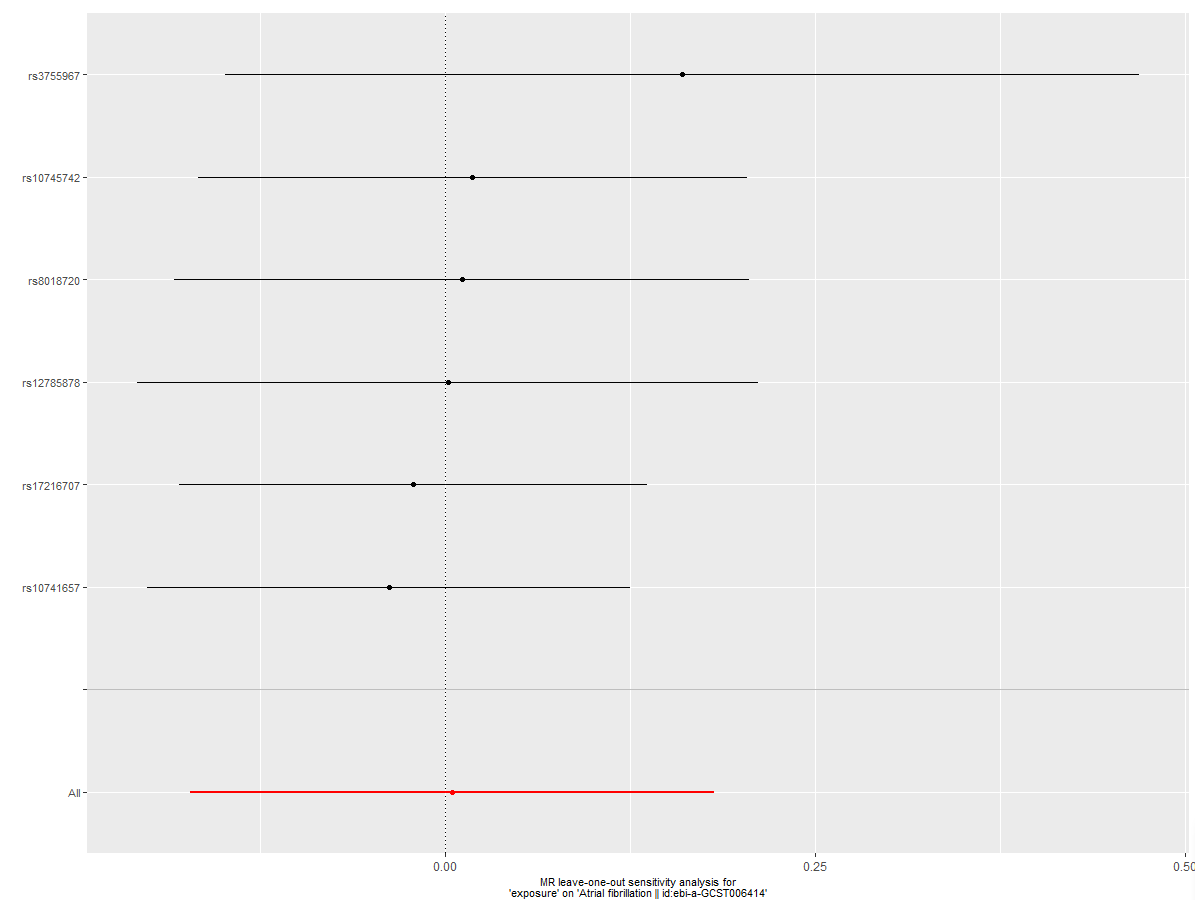


D. 25-hydroxyvitamin D levels and atrial fibrillation (Model 2)


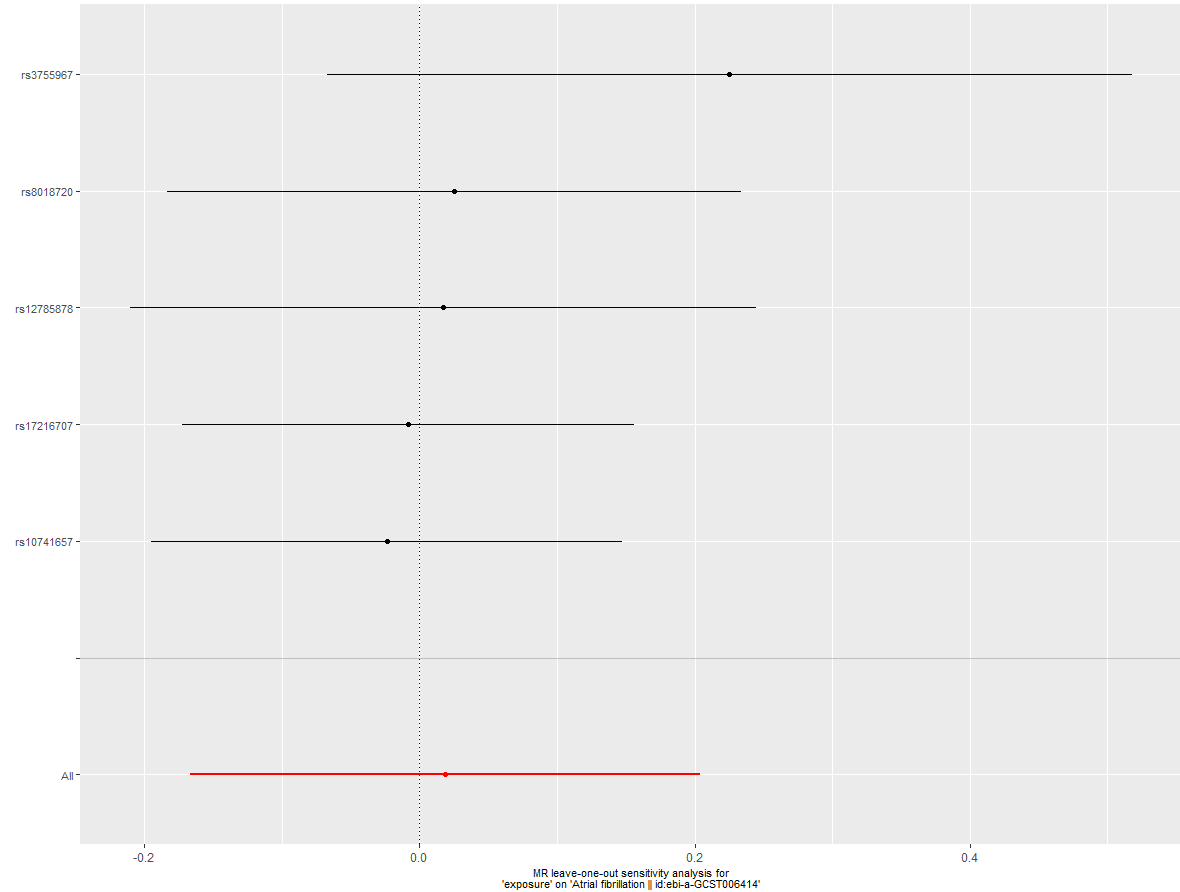


E. Atrial fibrillation and Vitamin D levels


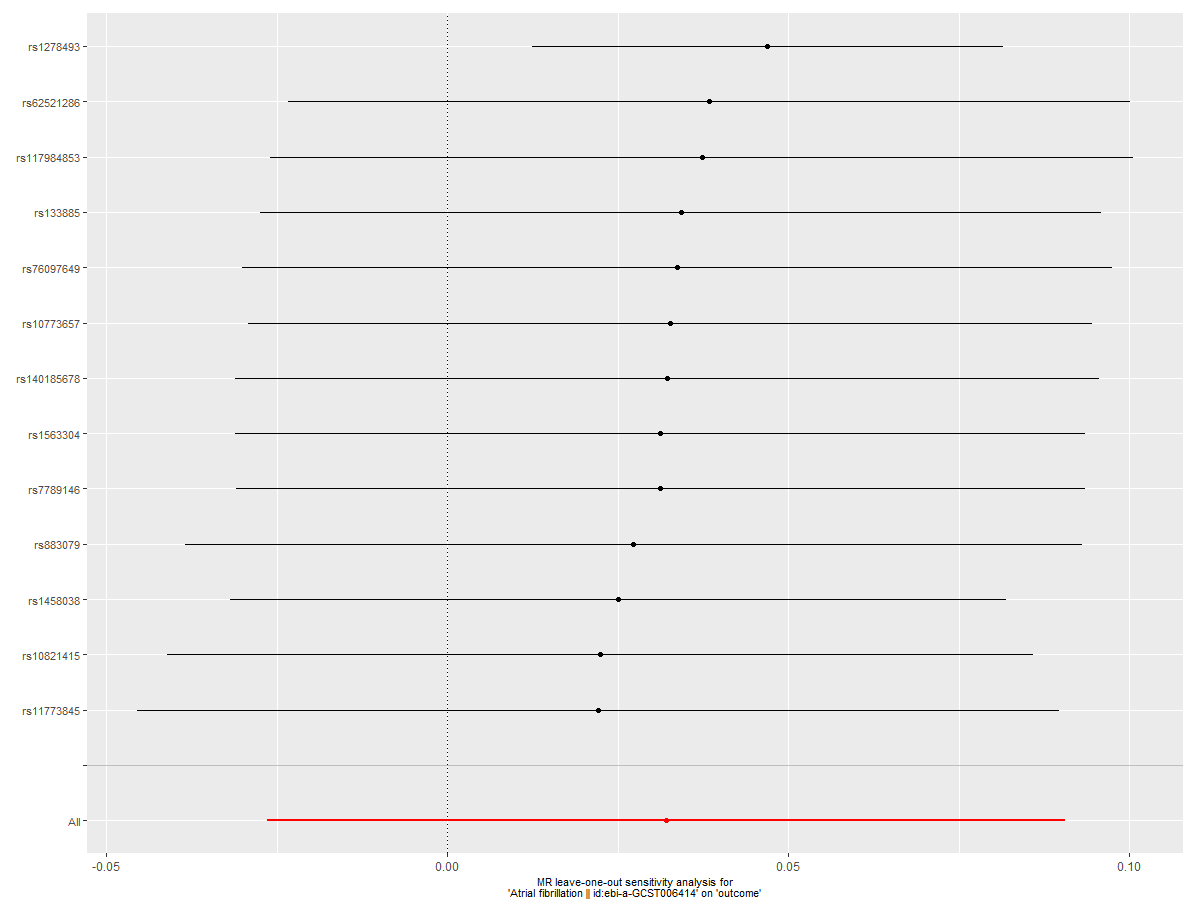


F. Atrial fibrillation and 25-hydroxyvitamin D levels


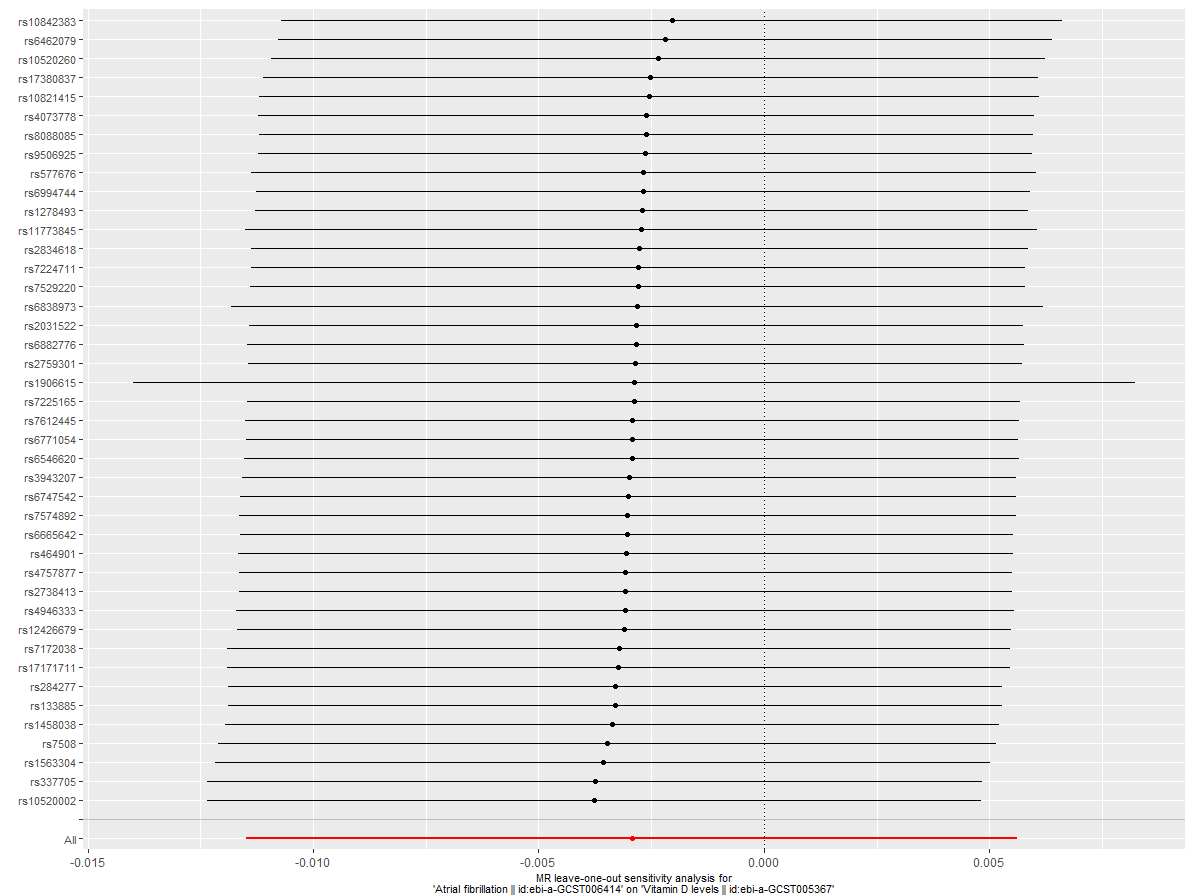

Supplement: Supplementary file 1 [file Data_Sheet_1.docx]
